# Proteomic Profiling of Extracellular Matrix Components from Patient Metastases Identifies Consistently Elevated Proteins for Developing Nanobodies That Target Primary Tumors and Metastases

**DOI:** 10.1158/0008-5472.CAN-22-1532

**Published:** 2023-04-26

**Authors:** Noor Jailkhani, Karl R. Clauser, Howard H. Mak, Steffen Rickelt, Chenxi Tian, Charles A. Whittaker, Kenneth K. Tanabe, Stephen R. Purdy, Steven A. Carr, Richard O. Hynes

**Affiliations:** 1 Koch Institute for Integrative Cancer Research, Massachusetts Institute of Technology, Cambridge, Massachusetts.; 2 Broad Institute of MIT and Harvard, Cambridge, Massachusetts.; 3 CAS Key Laboratory of Genomic and Precision Medicine, Beijing Institute of Genomics, Chinese Academy of Sciences, Beijing, China.; 4 Division of Gastrointestinal and Oncologic Surgery, Massachusetts General Hospital Cancer Center, Boston, Massachusetts.; 5 Camelid Immunogenics, Belchertown, Massachusetts.; 6 Howard Hughes Medical Institute, Chevy Chase, Maryland.

## Abstract

Metastases are hard to detect and treat, and they cause most cancer-related deaths. The relative lack of therapies targeting metastases represents a major unmet clinical need. The extracellular matrix (ECM) forms a major component of the tumor microenvironment in both primary and metastatic tumors, and certain ECM proteins can be selectively and abundantly expressed in tumors. Nanobodies against ECM proteins that show selective abundance in metastases have the potential to be used as vehicles for delivery of imaging and therapeutic cargoes. Here, we describe a strategy to develop phage-display libraries of nanobodies against ECM proteins expressed in human metastases, using entire ECM-enriched preparations from triple-negative breast cancer (TNBC) and colorectal cancer metastases to different organs as immunogens. In parallel, LC-MS/MS-based proteomics were used to define a metastasis-associated ECM signature shared by metastases from TNBC and colorectal cancer, and this conserved set of ECM proteins was selectively elevated in other tumors. As proof of concept, selective and high-affinity nanobodies were isolated against an example protein from this signature, tenascin-C (TNC), known to be abundant in many tumor types and to play a role in metastasis. TNC was abundantly expressed in patient metastases and widely expressed across diverse metastatic sites originating from several primary tumor types. Immuno-PET/CT showed that anti-TNC nanobodies bind TNBC tumors and metastases with excellent specificity. We propose that such generic nanobodies against tumors and metastases are promising cancer-agnostic tools for delivery of therapeutics to tumor and metastatic ECM.

**Significance:**

Nanobodies specific for extracellular matrix markers commonly expressed in primary tumors and metastases are promising agents for noninvasive detection of tumors and metastases and potential tools for targeted therapy.

## Introduction

Despite the promising successes of newer cancer therapies, the efficient detection and treatment of early or refractory solid tumors and occult metastases are still challenges that need to be addressed. There is a need to identify new targets, develop better-targeted therapies and detection strategies while reducing off-target effects and toxicities. Nanobodies against tumor-associated extracellular matrix (ECM) are promising tools to achieve this end.

While cancer cells remain the main target of most therapeutic approaches, the tumor microenvironment (TME), which comprises a substantial fraction of tumors provides a promising alternative target (1, 2). Targeting TME can help circumvent the challenges of heterogeneity, genomic instability, and development of drug resistance often observed in targeting cancer cells. Of particular interest are shared/common features of the tumor ECM, a major constituent of the TME (3–6). The ECM is a complex network of proteins that provide biochemical and biomechanical support to the neighboring cells in tissues (7, 8). ECM proteins play key roles in development, organ and tissue homeostasis, and regulate fundamental cellular processes such as cell proliferation, survival, migration, differentiation, and maintenance of tissue stiffness (9–11). ECM remodeling and deposition are characteristic of many diseases including cancer, fibroses, inflammatory and cardiovascular diseases and several ECM proteins play essential roles in these complex and progressive diseases (12–15). The ECM is very abundant, relatively stable and components of the ECM have low turnover rates (16). The ECM is much less prone to therapy-induced downregulation or mutations, which are frequently observed in cell surface receptors or signaling proteins expressed by cancer cells, potentially reducing the chances of developing resistance to targeted agents. Indeed, ECM plays a role in drug resistance (17–19) and is frequently upregulated in response to chemo and radiotherapy. Because most cancer-related deaths (>90%) are primarily caused by metastases, identifying new targets that are selectively and abundantly expressed in metastases can offer new routes to improving patient survival. Specifically, targeting the TME of the metastatic niche could allow more effective systemic therapy and reduce disease recurrence.

Recent advances in proteomic methods have helped delineate the detailed composition of the ECM in various healthy and diseased tissues; comprehensive characterizations of the matrisome (the entire set of ECM-associated proteins) have been done in recent years (5, 20, 21). These datasets have aided in the identification and characterization of key ECM players in tumor progression and metastasis and in identifying candidate ECM proteins that could be exploited as diagnostic and therapeutic targets (5). While the ECM composition of primary tumors has been relatively well studied (from both patients and mouse models) much less is known about the ECM of metastases. Some studies have profiled the ECM of multiple organ metastases in mouse xenograft models (22, 23); however the data on characterizing the ECM of patient metastases is limited (24), in large part because samples of human metastases are less readily obtained.

We previously developed a nanobody, NJB2, specific for a domain of fibronectin (FN-EIIIB), which is expressed selectively in many tumor types including triple-negative breast cancer (TNBC) and pancreatic ductal adenocarcinoma (PDAC) and their metastases (1). We showed by noninvasive *in vivo* imaging methods that NJB2 binds and detects tumors and metastases of different types (1). The current work aims to expand this approach to a broader range of targets. In this paper, we describe an improved strategy to identify ECM proteins selectively expressed in human metastases and isolate nanobodies targeting them. Several hurdles need to be cleared to identify a wider spectrum of ECM targets that could be targeted in human metastases. As mentioned, suitable metastatic samples are limited, compromising proteomic analyses. Furthermore, even when identified as candidate targets, ECM proteins are hard to purify for use as immunogens, given their insolubility and crosslinking; they are also frequently difficult to produce in recombinant form. These issues can be minimized by making the generation of nanobody libraries from metastases the starting point. By immunizing with the entire insoluble ECM-enriched fraction from human metastases one can evade the need to purify ECM proteins as immunogens and furthermore, can include in the immunogen all matrisome proteins found in those metastases. Subsequent generation of phage-display libraries from the immunized alpacas should then generate libraries of nanobodies against a wide variety of tumor-expressed ECM proteins. Parallel proteomic analyses of the same metastases used to generate the immunogens can help to identify which matrisome proteins are of most interest to acquire nanobodies from these libraries.

In this paper, we have taken this dual approach to generate libraries of nanobodies against ECM proteins expressed in human metastases and to identify (by proteomics) a 67-protein ECM signature associated with both human TNBC and colorectal cancer metastases to different organs. A large majority of these “metastasis-associated” signature proteins overlapped with matrisome signatures previously identified for primary tumors of different organs. Gene expression analyses of that signature and of the total matrisome in The Cancer Genome Atlas (TCGA) breast cancer samples confirms commonalities among the ECM proteins of multiple metastases to different sites and the ECM of the primary tumors, but not with the ECM of normal breast tissue. To provide an example of the potential of these phage-display nanobody libraries, we isolated monoclonal nanobodies against one exemplary protein from the ECM signature, tenascin-C (TNC), that is known to be expressed in multiple types of primary tumors and metastases and shows highly restricted expression in normal adult tissues (25). We developed nanobodies for use in *in vivo* PET/CT imaging and show that they recognize primary tumors and lung metastases with excellent clarity.

## Materials and Methods

### Patient samples

The acquisition of tissue samples was approved by the Institutional Review Boards (IRB) of the respective institutions. MIT IRB also approved the samples as exempt since anonymized. The liver metastasis samples from patients with colorectal cancer were obtained by Dr. Kenneth Tanabe (Massachusetts General Hospital, Boston, MA). Both patients had primary tumors in the colon that had been resected and had received chemotherapy prior to surgery and resection of liver metastases. Tissue samples were snap-frozen on dry ice directly after collection. Sample weights ranged from 0.7 to 1.3 g. The specimens were analyzed in accordance with a protocol approved by the Massachusetts General Hospital's IRB. These anonymized samples were removed for medical reasons unrelated to this project. The TNBC metastases to the lung and liver were obtained from Dr. Saraswati Sukumar (Johns Hopkins University, Baltimore, MD). The anonymized specimens were obtained from the Johns Hopkins School of Medicine's Autopsy program and were removed for medical reasons unrelated to this project. Samples were from end-stage patients who had undergone multiple treatments and were collected soon after their death. All samples were collected from patients who gave written informed consent. Duplicate samples from each patient were used for ECM enrichment.

### ECM enrichment

ECM enrichment was done using the CNMCS (Cytosol/Nucleus/Membrane/Cytoskeleton) compartment protein extraction kit (EMD Millipore) according to the manufacturer's protocol and using previously published methods (21, 24, 26). The kit provides buffers that allow the sequential depletion of proteins from different subcellular fractions. Briefly, using a bullet blender (Next Advance), between 50 and 150 mg of metastasis tissue were homogenized in the cytoplasmic buffer (“1”). The homogenized tissue was then treated sequentially with nuclear buffer (“2”), membrane buffer (“3”), cytoskeletal buffer (“4”), and the pellet was washed once again with the cytoplasmic buffer (“5”). Compared with the previously published protocols, this modified protocol has a single nuclear extraction step. The extracted fractions (1–5) were separated on SDS-polyacrylamide gradient gels, assessed by immunoblot with antibodies to proteins characteristic of different subcellular compartments. The removal of proteins from subcellular compartments leaves a final insoluble pellet, which is enriched for the ECM. The quality of enrichment was assessed by immunoblot. ECM proteins like fibronectin and collagen I are not extracted in the subcellular fractions. The fold enrichment varies between different tissue types but 8- to 10-fold enrichment is typical.

### ECM solubilization and digestion

LC-MS/MS analysis was done on two different regions (biological duplicates) of each patient metastasis sample. ECM solubilization and digestion was done using methods previously described (21, 26). Briefly, the ECM-enriched pellet was reduced and solubilized in a 100 mmol/L ammonium bicarbonate solution containing 8 mol/L urea and 10 mmol/L dithiothreitol. Cysteines were alkylated by addition of 25 mmol/L iodoacetamide and proteins were deglycosylated with enzyme PNGaseF (New England BioLabs). Proteins were digested with Lys-C (Wako Chemicals USA, Inc.) followed by Trypsin (Sequencing Grade, Promega). Peptides were acidified with trifluoroacetic acid, desalted using 30 mg HLB Oasis Cartridges (Waters Corp.), eluted with 60% acetonitrile, 0.1% trifluoroacetic acid and concentrated in a Speed-Vac. Details of NanoLC-MS/MS analysis, Protein/peptide identification and Label-free Relative Protein Quantitation are described in Supplemental Materials and Methods.

### Data availability

The original mass spectra for all experiments, and the protein sequence databases used for searches have been deposited in the public proteomics repository MassIVE (https://massive.ucsd.edu) and are accessible at ftp://MSV000089136@massive.ucsd.edu with username: MSV000089136; password: nanobodies. All other raw data are available upon request from the corresponding author.

### Phage-display library generation and selection of anti-TNC nanobodies

The phage-display libraries were prepared using methods described previously (1, 27). Nanobodies specific to the human TNC protein were selected by panning Library B against human recombinant TNC protein (Sigma). Phage panning and nanobody selection were done using previously described methods (1, 27). Briefly, 100 μg of TNC was buffer-exchanged into the modification buffer (100 mmol/L sodium phosphate, pH 7.4, 150 mmol/L NaCl). The protein was biotinylated on primary amines using the Chromalink NHS-biotin reagent (Solulink) according to the manufacturer's protocol. For panning, we washed 100 μL of MyOne Streptavidin T1 Dynabeads (Life Technologies) and blocked them with 2% (wt/vol) BSA (Sigma) in PBS for 1 to 2 hours. The beads were incubated for 30 minutes with 20 μg of biotinylated TNC in 2%BSA/PBS at 25°C and washed 3 times with PBS. The beads were mixed with 200 μL of prepared phage (10^14^ pfu/mL) in 2% wt/vol BSA in PBS and incubated for 1 hour at room temperature. The beads were washed 15 times with PBS containing 0.1% Tween-20. The phage were eluted from the beads by the addition of a saturated culture of 500 μL ER2738 *E. Coli (*New England BioLabs) for 15 minutes at 37°C and then with 500 μL of 0.2 mol/L glycine, pH2.2 for 10 minutes at 25°C. The glycine elution was neutralized with 1 mol/L Tris pH 9.1, pooled with the ER2738 *E. Coli* culture and plated on 2YT plates supplemented with glucose (2% wt/vol), tetracycline (5 μg/mL), and ampicillin (10 μg/mL) and incubated overnight at 37°C. This library was used for the second round of panning, which involved some modification—2 μg of biotinylated TNC was used as bait protein and incubated with 2 μL of phage (10^14^ pfu/mL), displaying the first round of panned library for 15 minutes at 37°C. After the second round of panning, an ELISA was done to identify positive clones using methods previously described (1, 27).

### Mouse strains and animal models

All mice used for 2-photon imaging and PET/CT imaging experiments were maintained at the mouse facility in MIT's Koch Institute for Integrative Cancer Research in accordance with protocols approved by the MIT Institutional Animal Care and Use Committee (IACUC). NSG (NOD/SCID/IL2Rg-null) mice were purchased from Jackson Laboratory. Alpacas (*Vicugna pacos*) were maintained, immunized, and bled at a farm in Massachusetts in accordance with a protocol authorized by the University of Massachusetts (Amherst) Veterinary School's IACUC and the MIT IACUC.

### *In vivo* tumor formation and metastasis

For induction of orthotopic primary tumors, NSG mice were injected in their fourth mammary fat pads (MFP) with 1×10^6^ LM2-TGL-Zsgr cells and allowed to grow for 3 to 3.5 weeks depending on the experiment. For the pulmonary metastasis model, NSG mice were injected in the lateral tail vein with 0.4×10^6^ to 0.6×10^6^ LM2-TGL-Zsgr cells and the metastases were allowed to grow and develop for 3 weeks. IVIS imaging was used to monitor disease progression.

### Bio-layer interferometry

Bio-layer interferometry (BLI) was done using a ForteBio Octet RED96 bio-layer interferometer (Pall ForteBio) to determine the affinities of the hTNC-specific nanobodies to full-length human TNC protein. Streptavidin-coated BLI biosensor tips (Forte Bio) were soaked in the assay running buffer consisting of PBS supplemented with 0.05% Tween-20 and 1% recombinant human albumin (Sigma) for 20 minutes. To capture the nanobodies the biosensor tips were immersed in 100 nmol/L solution of biotinylated nanobodies NJT3, NJT4, or NJT6. The nanobody-loaded tips were then dipped into wells containing different concentrations of purified hTNC protein (Sigma-Aldrich). The data were reference-subtracted and the association and dissociation rate constants were analyzed using the ForteBio data analysis software (V8.2) using a 1:1 binding model and global fit analysis.

### PET/CT imaging and analysis

Immuno-PET imaging was done by injecting 68.7 ±11 uCi of ^64^Cu-labeled nanobodies in 100 μL of sterile PBS into the lateral tail vein of anesthetized mice (2% isoflurane mixed in oxygen). Mice were woken up and allowed to move around in their cages for probe uptake and imaged 120 minutes after probe injection. ^18^F-FDG imaging was done by injecting ∼100 uCi of ^18^F-FDG in 100 μL of sterile PBS into the lateral tail vein of mice that had been fasted for 10 to 12 hours. During and after injection, mice were kept unconscious under anesthesia (2% isoflurane mixed in oxygen) to reduce probe uptake into muscles and imaged 60 minutes after probe injection. Body temperature was maintained using controlled heating pads. For both immuno-PET/CT and ^18^F-FDG PET/CT imaging, mice were imaged with a static PET scan for 10 minutes followed by a 1.5-min CT scan for anatomic reference using a G8 PET/CT preclinical, small-animal scanner (PerkinElmer). Images were processed using the manufacturer's automatic image reconstruction software. Image visualization was done using the VivoQuant software version 3.5 (Invicro). Dicom files were loaded into the VQ software and the PET/CT data were co-registered. PET radioactivity values were decay-corrected and converted into PET-standardized uptake values (SUV). All images are represented as maximum-intensity projections of all slices and scaled to PET SUV, unless otherwise indicated. PET/CT images of mice were viewed side-by-side to allow comparison between controls and disease-bearing mice. Details of biodistribution analysis are described in Supplementary Materials.

## Results

### ECM enrichment-based strategy to develop nanobodies against metastasis-associated ECM proteins

**Figure 1.**
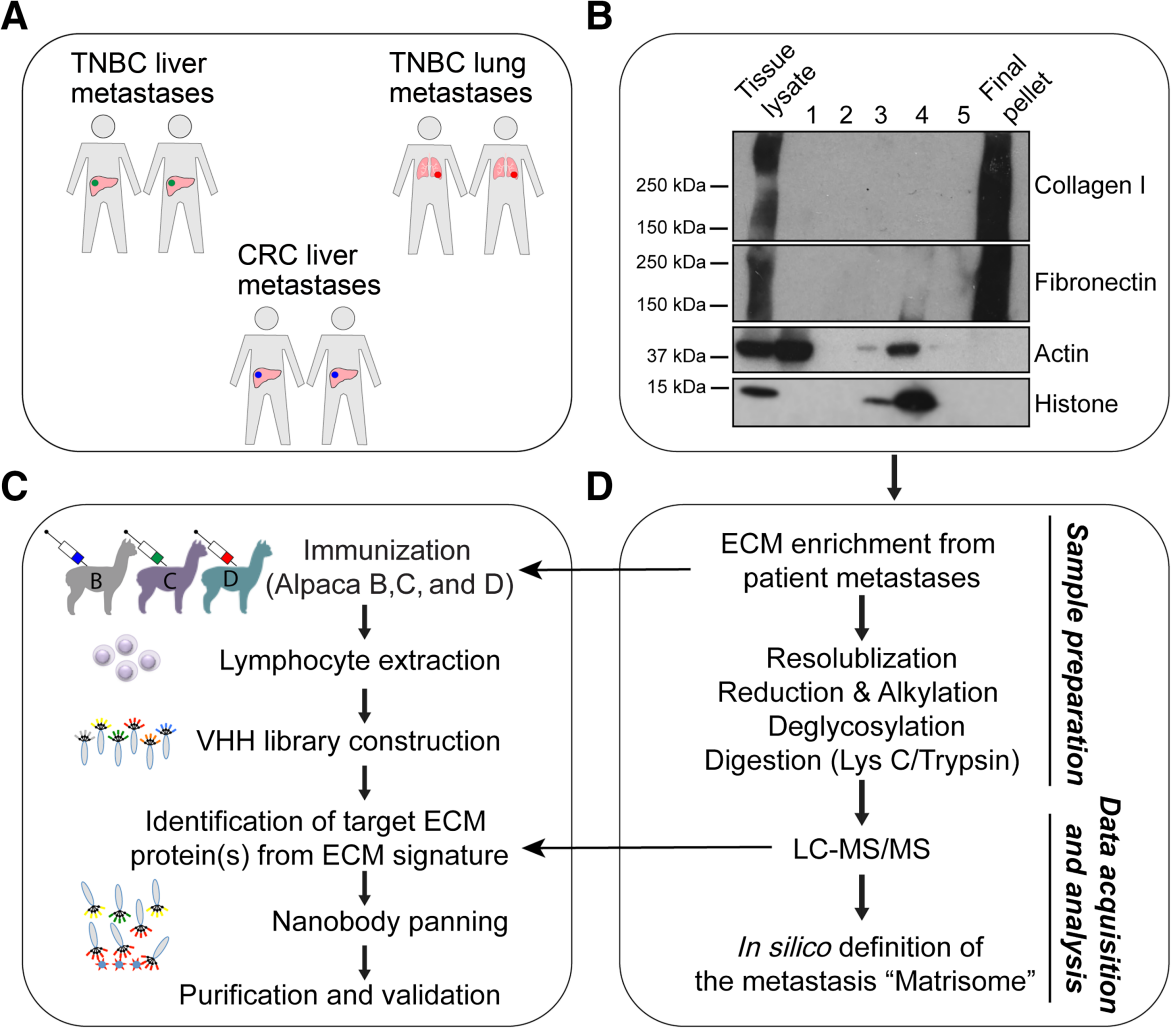
Schematic of ECM enrichment and proteomic analysis of patient metastasis samples for matrisome nanobody library construction and panning. **A,** Patient metastasis samples used for ECM enrichment and proteomics for identification of ECM proteins/signatures associated with patient metastases. CRC, colorectal cancer. **B,** Immunoblots showing sequential extraction steps (1–5; see Materials and Methods) to obtain the final insoluble ECM-enriched pellet. **C,** Simplified schematic of nanobody library construction and isolation of nanobodies against ECM proteins identified from proteomics signatures. (See also Supplementary Fig. S1 for details on immunogen and immunization schedule.) **D,** Simplified schematic of LC-MS/MS–based proteomics pipeline to characterize the matrisome of the human metastases.

An outline of our experimental strategy to develop nanobodies against multiple proteins associated with metastases is indicated in Fig. 1. We obtained human patient samples of TNBC metastases to the liver and lungs and colorectal cancer metastases to the liver (*N* = 2 patients for each sample type; Fig. 1A). This was followed by ECM enrichment from each metastatic tissue using a modified subcellular fractionation protocol (see Materials and Methods) and the ECM enrichment was verified by immunoblot (Fig. 1B).

These enriched ECM samples were used to immunize alpacas for generation of three ECM-specific phage-display nanobody libraries (libraries B, C, and D), one for each metastatic location and tumor type (Fig. 1C; Supplementary Fig. S1; Materials and Methods and Supplementary Methods). To capture the potential diversity of ECM proteins across different patient metastases, we pooled the ECM-enriched preparations from two different patients (for each metastasis site) and immunized alpacas with these combined mixtures (Supplementary Fig. S1). Pre-immune and post-immune sera were collected and tested to assess immune responses to ECM-enriched pellets using Western blots. Following library construction in M13 phage-display vectors, we observed diversity in the range of 10^7^ to 10^8^, which is within the expected diversity of immune nanobody libraries (Supplementary Fig. S1; ref. 28). Maximum diversity was observed in the 3 CDR regions. Nanobodies obtained following phage panning were expressed, purified, and validated by methods previously described (1, 27, 29).

### Matrisome signatures of TNBC and colorectal cancer metastases to different organs

In parallel, duplicate samples of the ECM-enriched fractions from each tissue were analyzed using LC-MS/MS followed by *in silico* definition of the matrisome composition (Fig. 1D). For the LC-MS/MS analysis, ECM enrichment was done from two different pieces of the same tumor to allow assessment of intra-lesion heterogeneity in ECM and ECM-associated proteins. We found that biological replicates had varying degrees of overlap; 96% for TNBC Liv met 1, 72% for Liver Met 2, 91% for TNBC Lg 1, 87.5% for TNBC Lg 2 and ∼85% for both the colorectal cancer liver met samples.

Following LC-MS/MS characterization of the ECM-enriched pellets; we compared the contributions of matrisome and non-matrisome proteins (see Supplementary Materials and Methods). Peptides from matrisome proteins contributed the great majority of the precursor-ion intensity; typically > 90%, indicating successful ECM enrichment (Supplementary Fig. S2A), although TNBC liver metastases were somewhat less well enriched (64.8% and 74.2%). Each ECM preparation yielded ∼110 to 170 identified matrisome proteins and the numbers of proteins in the different matrisome categories are shown in Supplementary Fig. S2B and Supplementary Table S1. The distributions of numbers of proteins in the different matrisome categories across the samples were similar, suggesting similar levels of complexity.

**Figure 2.**
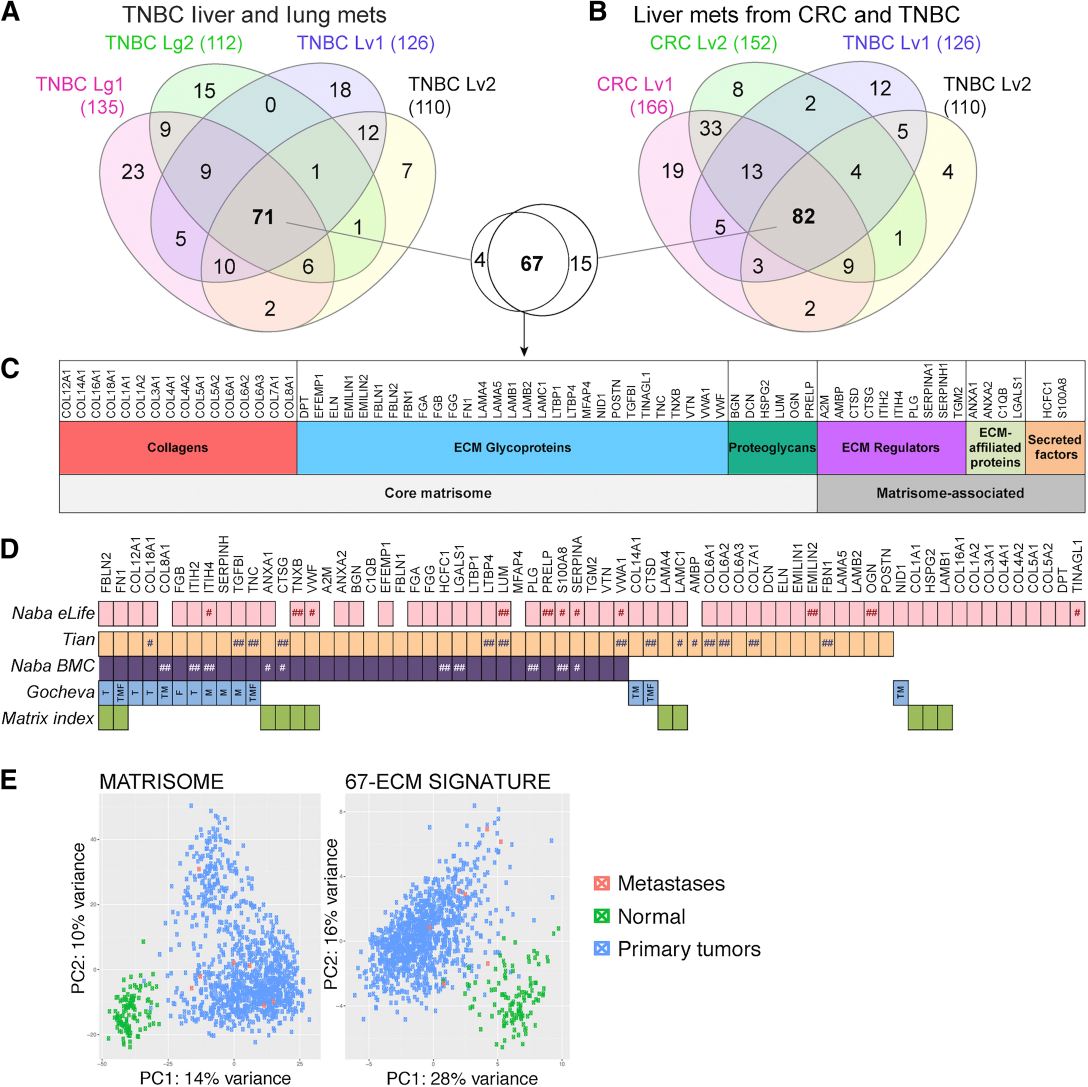
Proteomic analysis of ECM-enriched fractions identifies signatures associated with human TNBC and colorectal cancer metastases to different organs. **A,** Venn diagram showing the overlap of matrisome proteins identified from TNBC metastases from four different patients to two different metastatic sites (lungs and liver). (See Supplementary Table S1 for full lists of proteins.) **B**, Venn diagram showing the overlap of matrisome proteins identified from TNBC and colorectal cancer metastases to the liver from four different patients. (See Supplementary Table S1.) **A** and **B**, Inset, Venn diagram showing the 67-protein “metastasis-associated ECM signature” shared by all patient samples. **C,** Figure lists the identities of the 67 proteins in the metastasis-associated signature and their matrisome categories. **D,** Overlap of the metastasis-associated signature with ECM signatures identified in previously published literature of mammary carcinoma (Naba eLife), PDAC (Tian; ref. 30), colorectal cancer (Naba BMC), lung adenocarcinoma (Gocheva; ref. 31), and ovarian cancer (Matrix index). Naba eLife, pink boxes are proteins detected in both LM2- and 231-derived tumors; #, detected in LM2 only; ##, detected in 231 only. Tian and colleagues (30), orange boxes are proteins significantly overrepresented in PanIN and PDAC; #, significantly overrepresented in PanIN only; ##, significantly overrepresented in PDAC only. Naba BMC, purple squares represent proteins with a 10-fold higher abundance in colon tumors compared with normal colon; #, 10-fold higher abundance in liver metastases compared with normal liver; ##, 10-fold higher abundance in both primary tumors and liver metastases compared with corresponding normal tissues. Gocheva and colleagues (31), proteins that were significantly abundant in the following disease tissues are indicated as T, tumor; M, lung metastases; F, lung fibroses. **E,** PCA plots showing expression of all matrisome genes and the 67-ECM signature from RNA sequencing data derived from TCGA-BRCA dataset. Both plots show a clear separation between the normal (green) and primary tumors (blue) and a co-clustering of the metastases with the primary tumor cluster. Lg1 and 2 refer to lung metastases from patient 1 and 2; Lv1 and Lv2 refer to liver metastases from patient 1 and 2. CRC, colorectal cancer.

Potential target proteins were subsequently identified from the ECM signatures defined by the LC-MS/MS analysis. To understand inter-patient heterogeneity we compared the lists of identified ECM proteins across different patient samples. The Venn diagrams in Fig. 2A and B indicate proteins that are unique to a particular metastatic lesion or shared across different lesions. In the comparison of TNBC lung metastases, we found that, between the 2 patients, TNBC Lg1 (135 matrisome proteins) and TNBC Lg2 (112 matrisome proteins), 95 ECM proteins were shared. Similarly, in patients with TNBC liver metastases (Patient Lv1 with 126 proteins and patient Lv2 with 110 proteins) 94 proteins were shared. To identify ECM signatures associated with TNBC metastases to different sites we compared the proteins that were commonly present across all 4 patients and the 2 metastatic sites of the lungs and liver (Fig. 2A); 71 proteins were present in all the samples.

We were also interested in identifying ECM signatures associated with metastases from different primary sites to the common site of the liver and we overlaid the data from TNBC liver metastasis samples with colorectal cancer liver metastases (colorectal cancer Lv1 and Lv2). Here we found that 82 proteins were present in all 4 patients, suggesting a common liver metastasis signature across different primary tumor types (TNBC and colorectal cancer) to the same metastatic site (Fig. 2B). 67 matrisome proteins were present in all six patient metastasis samples (Fig. 2C).

We compared this 67-protein “metastasis-associated” signature with previously published ECM datasets for human TNBC mammary carcinoma xenografts, “Naba eLife” in Fig. 2D (26). 60 proteins from our signature were also expressed in tumors derived from either cell lines MDA-MB-231 or LM2 or both. Because the “Naba eLife” study did not include profiling of normal breast tissues, to identify proteins that are more abundant in diseased tissues compared with normal, we examined 4 other signatures including (i) human PDAC stages, “Tian” (30); (ii) human colorectal cancer and colorectal cancer liver metastases, “Naba BMC” (24); (iii) the “Matrix-index” a human ovarian omental metastasis-based signature (4); and (iv) murine lung adenocarcinoma, “Gocheva” (31). In Tian and colleagues that describes ECM signatures for human PanIN and PDAC stages compared with normal human pancreas, 40 proteins from our signature were significantly overrepresented in both PanIN and PDAC stages, 11 were significantly overrepresented only in the PDAC stage (Fig. 2D, ##) and 3 proteins only in the PanIN stage (Fig. 2D, #). For the human colorectal cancer and colorectal cancer liver metastasis signatures, we reanalyzed the data presented in Naba and colleagues and report here proteins that had a 10-fold or higher abundance in disease tissues (tumors or metastases), compared with the corresponding normal tissues. We found the abundance of 26 proteins from our signature to be 10-fold or more higher in the primary tumors compared with the normal colons, 3 proteins were higher in liver metastases compared with normal livers (Fig. 2D, #) and 7 proteins were higher in both tumors and liver metastases (Fig. 2D, ##) compared with their corresponding normal tissues. Thus, 36/67 proteins in our signature were significantly elevated in colon tumors and/or metastases. In Gocheva and colleagues that characterizes the matrisome of murine lung adenocarcinoma, lymph node metastases, lung fibrosis and normal murine lung, 53 proteins from our signature were expressed in all samples including normal lung. However, the expression of 14 of these was significantly higher in diseased tissue (tumor, lymph-node metastases or fibrosis) compared with normal lung (Fig. 2D). We also compared our signature with the “matrix index”; a signature of 22-matrix proteins derived from ovarian cancer metastases that is associated with shorter survival in ovarian cancer and 12 other cancer types, and found that 11 of the 22 ECM proteins were shared with our signature.

In summary, 58 of the 67 proteins from our “metastasis-associated” signature were overrepresented in at least 1 of the 4 published signatures. FN1 and FBLN2 were expressed in human mammary xenograft tumors and also overrepresented in disease tissues in all 4 signatures. Others, such as COL18A1, TGFBI, FGB, ITIH2, ITIH4, and TNC among others, were overrepresented in 3 of 4 signatures and also expressed in mammary carcinoma xenografts. This analysis confirms that many proteins from our metastasis-associated signature are not only expressed in primary tumors of the breast, but also are overrepresented compared with normal controls in tumors and metastases derived from multiple different organs and cell types.

Because the proteomics-based matrisome analyses in the aforementioned datasets are limited to a few patient samples, we sought to evaluate the expression of the “metastasis-associated” signature in a larger patient sample set. We compared the expression of all genes in the matrisome and the 67-ECM signature in the large TCGA-BRCA (breast cancer) data set, which consists of 1,052 primary breast tumor samples, 112 normal breast tissue samples and 7 metastases samples. The first 2 principal components that capture the most variance are shown for the total matrisome and for the 67-ECM signature (Fig. 2E). The principal component analysis (PCA) plots of the samples show a clear separation between the primary breast cancer tumors and the normal breast tissue samples. In both analyses, the metastasis samples co-clustered with the primary tumor cluster and were well separated from the cluster of normal breast tissue. A similar analysis comparing 120 primary breast tumors and 26 metastases was done using data from the metastatic breast cancer project (https://www.mbcproject.org/). Although this dataset lacks normal tissue samples, PCA for the matrisome genes and the 67-ECM signature, comparing the primary tumors and metastases showed that the metastases co-clustered with the primary tumors and did not form a distinct cluster (Supplementary Fig. S2C). Both these analyses suggest that the ECM of mammary tumor–derived metastases is closer to the ECM of the primary tumors than to that of the normal breast tissues, as expected (5, 24).

**Figure 3.**
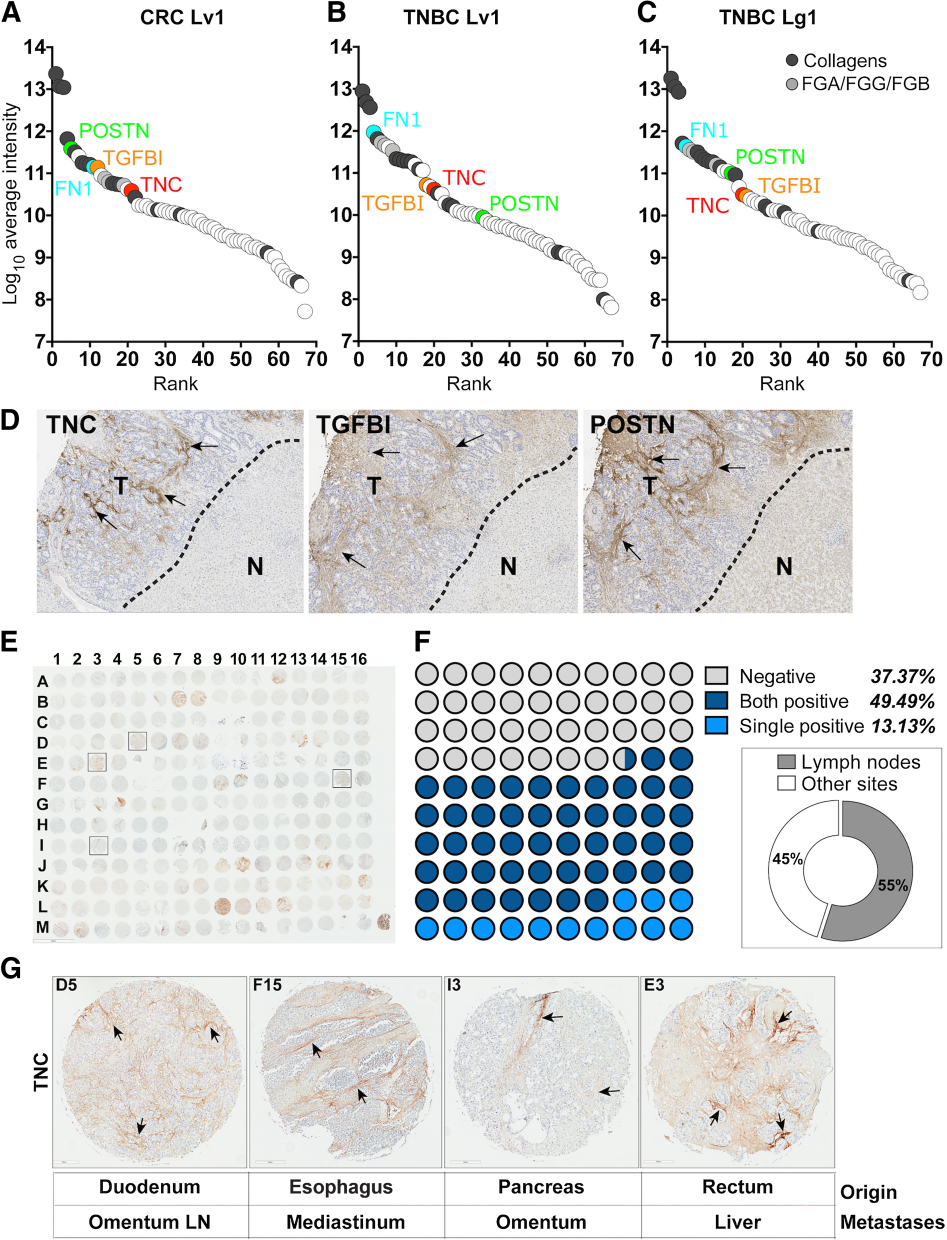
**A-C,** Sixty-seven proteins present in the metastasis-associated ECM signature shared among the 6 patient samples, ranked on the basis of their intensities (mean of the two duplicate ECM-enriched samples). Plots for colorectal cancer liver metastasis patient 1 (**A**), TNBC liver metastasis patient 1 (**B**), and TNBC lung metastasis patient 1 (**C**). Some ECM proteins are marked including FN (blue) and those tested for IHC in **D**. TNC, red; TGFBI, orange; POSTN, green. See also Supplementary Table S2 with rankings. **D,** IHC on a colorectal cancer liver metastasis sample shows that expression of TNC, TGFBI, and POSTN is restricted to the metastatic lesion (T) and absent from adjacent normal liver tissue (N). Arrows, regions of positive fiber-like staining. See also Supplementary Fig. S3. **E,** Testing expression of TNC in a multi-organ patient metastatic tissue array by IHC, with TNC-specific monoclonal antibody. Array contains two biopsy samples from each patient (*N* = 104). **F,** Scoring of TMA for stromal positivity of TNC, indicating patients that were negative (gray), patients with a single positive biopsy (light blue), and patients with both biopsies positive (dark blue). Inset shows percentage of positive biopsies that were metastases from lymph nodes at the target organs. **G,** Representative examples of metastases from **E** that were positive for TNC expression from four different primary tumor types (origin) to different metastatic sites. Arrows, positive fiber-like staining. LN, lymph node. CRC, colorectal cancer.

We next ranked the ECM signature proteins based on their abundance in each sample and found that, unsurprisingly the most abundant ECM proteins in all samples were collagens (Fig. 3A–C; Supplementary Table S2). However, among the other most abundant ECM proteins shared across all samples were components of basement membranes, such as laminins, HSPG2, collagen IV and VI, as well as fibrinogen chains (FGA, FGB, FGG) and ECM glycoproteins such as FN1, POSTN, VTN, FBN1, EMILIN1, TGFBI, and TNC (Supplementary Table S2). We have already isolated and deployed nanobodies against a recombinant fragment of FN1 in previous studies (1).

To explore further these proteins as potential tumor-specific targets, we tested their expression in one of the patient colorectal cancer liver metastasis samples by IHC using available verified and validated antibodies (32) and compared their expression in the adjacent normal tissue. The proteins tested included TNC, HSPG2, POSTN, TGFBI, and EMILIN1. We found that, among some of the most abundant glycoproteins on our list, the expression of TNC, TGFBI, and POSTN was restricted to the metastatic lesion (Fig. 3D). Others such as EMILIN1 and HSPG2 were expressed in both tumor and adjacent normal tissue (Supplementary Fig. S3A) and, thus, unsuitable for selective targeting of malignant tissue. Thus, TNC, TGFBI, and POSTN appear as reasonable initial candidates for disease-selective targets.

### TNC as an example target in multiple metastatic cancers

We elected to use TNC as a representative example to demonstrate and further explore the strategy of using complex ECM-enriched immunogens to focus on promising metastasis-expressed targets. TNC is a large ECM glycoprotein, which is known to show restricted expression in adult tissue, but is expressed during embryogenesis, wound healing, chronic inflammation and cancer (25). The role of TNC in tumor progression and metastasis is well established (33, 34). TNC is expressed in many primary tumor types and proteomic analyses have shown that TNC is a very abundant ECM protein in the stroma of solid tumors including breast cancer, PDAC and lung cancer (26, 30, 31). As demonstrated above, TNC was also one of the more abundant tumor-specific ECM proteins identified in the “metastasis-associated” signature and was overrepresented in diseased tissues compared with normal in 3 of the 4 signatures analyzed in Fig. 2D. This prompted us to investigate the potential of TNC as a promising example of a target in multiple metastatic cancers. To test its expression in metastases from different primary tumor types and at different metastatic sites, we stained a tissue microarray (TMA) of multiple human metastases for the expression of TNC protein (Fig. 3E). This human multi-organ metastatic tissue array had samples derived from 104 patients with 2 biopsies per patient. Remarkably, we found ∼63% of the patients expressed TNC in their metastases (Fig. 3F). While nearly half (49.49%) of the patients were positive for TNC expression in both biopsy samples, ∼13% were found to express TNC in only 1 biopsy. Around half of the positive biopsies were metastases to the lymph nodes of the target sites (Fig. 3F, inset). Among the positive biopsies were metastases to 25 different metastatic sites and these were derived from tumors that originated in 20 different primary sites (Fig. 3G; Supplementary Fig. S3B). These data show that TNC is widely expressed across various metastatic sites derived from multiple tumor types. Its restricted expression in normal human tissues (25), abundance in the ECM signatures of the human TNBC and colorectal cancer metastases and its wide expression not only in multiple primary tumor types but also across multiple metastatic sites suggested that TNC could be an exemplary candidate for nanobody-based imaging and therapeutic applications.

### Generation and characterization of hTNC-specific nanobodies

**Figure 4.**
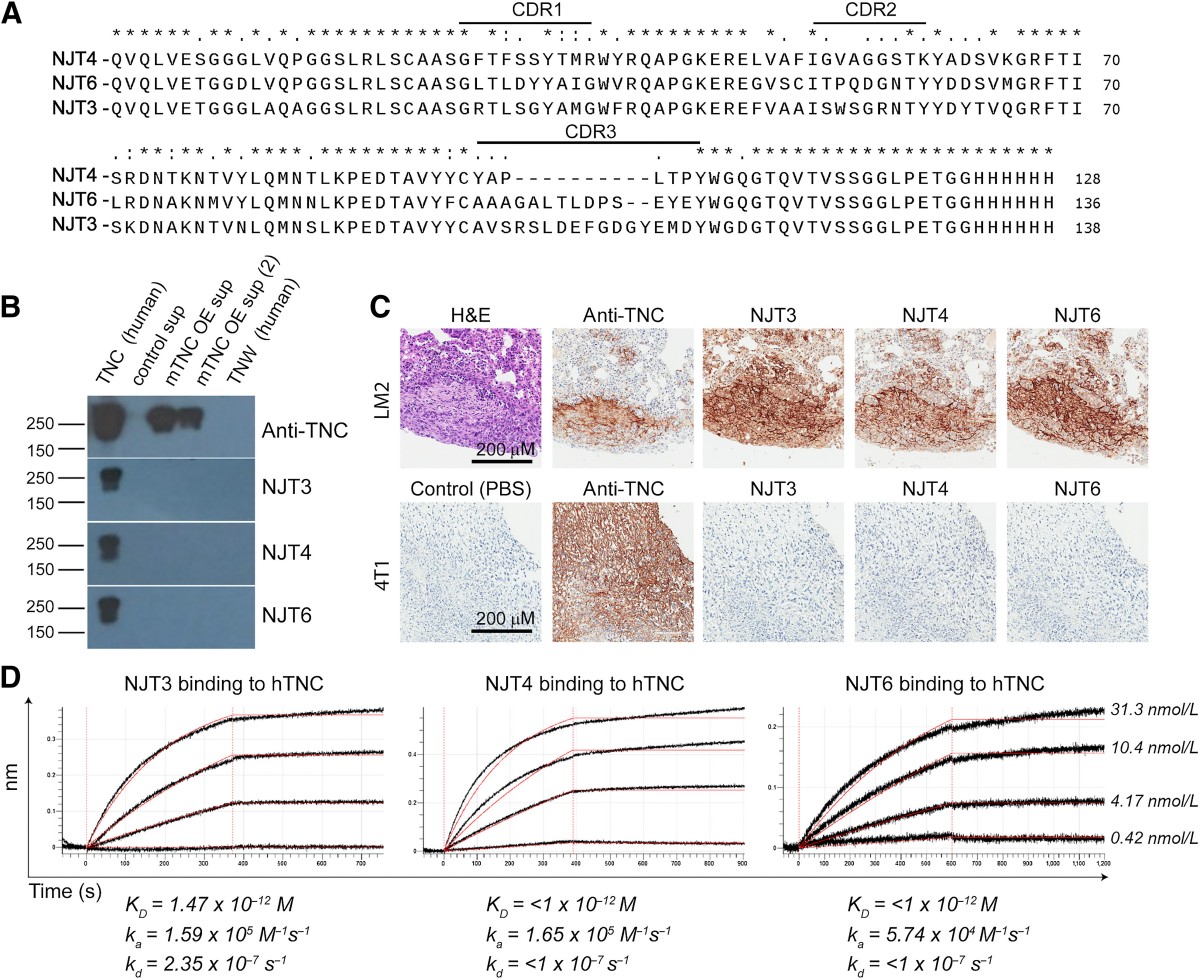
TNC-specific nanobodies recognize human TNC with subnanomolar affinity. **A,** Clustal W–based sequence alignment of anti-human TNC nanobodies NJT3, NJT4, and NJT6. CDR, complementarity-determining region. **B,** Immunoblot showing specificity of TNC-specific nanobodies to human TNC protein. The nanobodies do not recognize mouse TNC in supernatants from TNC-overexpressing (OE) cell lines, supernatant from control cells (control sup), or human TNW. **C**, Anti-TNC nanobodies recognize human TNC in IHC on tissue sections of lung metastases derived from human TNBC cell line LM2. They did not recognize mouse TNC on tissue sections of lung metastases derived from murine TNBC cell line 4T1. The control rabbit monoclonal α-TNC antibody recognizes both human and mouse TNC. **D,** Dissociation constants (K_D_) of α -TNC nanobodies to immobilized human TNC protein measured using BLI. KD, ka, and kd values are listed. Black curves, original data; red curves, curve fitting. H&E, hematoxylin and eosin.

Accordingly, we used the phage-display nanobody library “B” raised against ECM of colorectal cancer metastases to the liver, to pan against full-length human TNC protein using methods described previously (1, 27). ELISA-positive clones were expressed with a C-terminal sortase tag, which allowed site-specific addition of biotin or fluorophores for *in vitro* validation assays. Our three selected clones (NJT3, NJT4, and NJT6) had distinct CDR 1, 2, and 3 sequences (Fig. 4A). Although human and murine TNC share a high sequence identity, these nanobodies, which are each monoclonal, recognized human TNC protein by immunoblot, but did not bind mouse TNC or human tenascin-W (TNW; Fig. 4B). The control rabbit monoclonal antibody (anti-TNC, Abcam) detected both human and mouse TNC protein. The three nanobodies detected and bound TNC in the ECM of lung metastasis samples from NSG mice injected via the tail-vein with LM2 human TNBC cells as shown by IHC (Fig. 4C). The nanobodies did not detect TNC in lung metastasis samples derived from BALB/c mice injected with murine TNBC cell line 4T1. In comparison, the control anti-TNC rabbit monoclonal antibody recognized both human and mouse TNC (Fig. 4C). The binding affinities of the nanobodies to human TNC protein were measured by BLI. All three nanobodies bound full-length human TNC protein with picomolar or subpicomolar affinities (K_D_) and very low off rates as indicated in Fig. 4D demonstrating high affinity for its target antigen.

### hTNC-specific nanobodies selectively bind the ECM of primary tumors and discrete metastases in a human TNBC xenotransplant model

**Figure 5.**
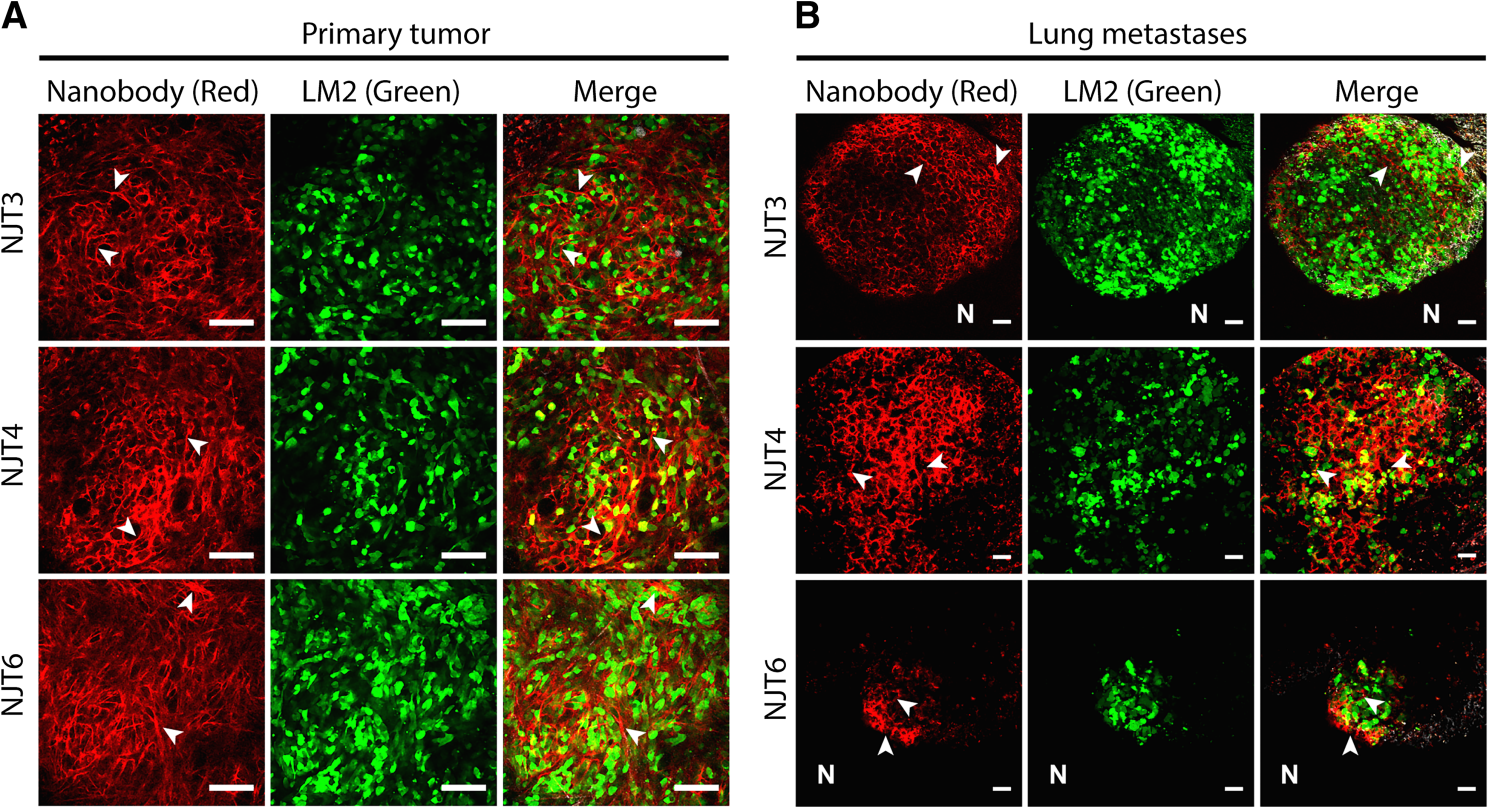
Anti-TNC nanobodies recognize the ECM of primary tumors and lung metastases in a TNBC model. α-TNC nanobodies detect the ECM of tumors and lung metastases derived from LM2 TNBC cells. **A** and **B,** NSG mice bearing orthotopic tumors (**A**) or lung metastases (**B**) derived from LM2-TGL-ZsGreen cells were injected intravenously with Alexa-647 labeled nanobodies. Two hours after injection, indicated organs were resected and imaged by two-photon microscopy. Representative images are shown as an overlay of different channels. α -TNC nanobodies bound to the ECM of both primary tumors and lung metastases (arrowheads) but not normal tissue ECM (N). Scale bars, 100 μm.

To test their ability to bind the ECM of tumors and metastases *in vivo*, the three nanobodies, NJT3, NJT4, and NJT6, were each tagged at their C-termini with fluorophore Alexa-647 using sortase-mediated tagging. The tagged nanobodies were injected via the tail vein into NSG mice that had orthotopic primary tumors derived from LM2-TGL-ZsGreen cells. Two-photon imaging showed signals from fibrillar structures (white arrowheads) surrounding the Zs-green-expressing tumor cells, indicating that all three nanobodies were able to bind TNC fibrils in the ECM of these tumors (Fig. 5A).

These mice also had discrete lung metastases that had seeded from the orthotopic site. To test whether the nanobodies were able to bind small discrete lung metastases, we also imaged the lungs from these mice. In mice injected with the labeled nanobodies, clear ECM fibrils were seen surrounding the Zs-green-positive metastatic cells suggesting that all three nanobodies were able to bind and detect small metastases. The size of the metastases ranged from a few hundred microns to a few millimeters, indicating that the nanobodies can bind micrometastases, which are hard to detect and treat (Fig. 5B). As expected, none of the nanobodies bound to the adjacent normal mouse lung ECM (“N”) or the ECM of normal lungs resected from control mice injected with labeled nanobodies (Supplementary Fig. S4).

### Noninvasive detection of tumors and lung metastases by immuno-PET/CT using ^64^Cu-labeled nanobodies specific for human TNC

To test these nanobodies as tools for *in vivo* imaging and potential therapeutic applications we assessed their clearance in NSG mice using ^64^Cu-labeled nanobodies (see Supplementary Methods). We found that 2 hours after injection, in mice injected with ^64^Cu-NJT3 and ^64^Cu-NJT4, about half the injected nanobody had cleared but nearly half (∼53%) of the activity was still retained (Supplementary Fig. S5). However, in comparison, in mice injected with ^64^Cu-NJT6, ∼90% of the nanobody had cleared from the mouse within 2 hours of nanobody injection (Supplementary Fig. S5). This was significantly lower than the retention observed in mice injected with ^64^Cu-NJT3 and ^64^Cu-NJT4 and was comparable with the values observed for ^64^Cu-NJB2, a FN-EIIIB-specific nanobody (1).

**Figure 6.**
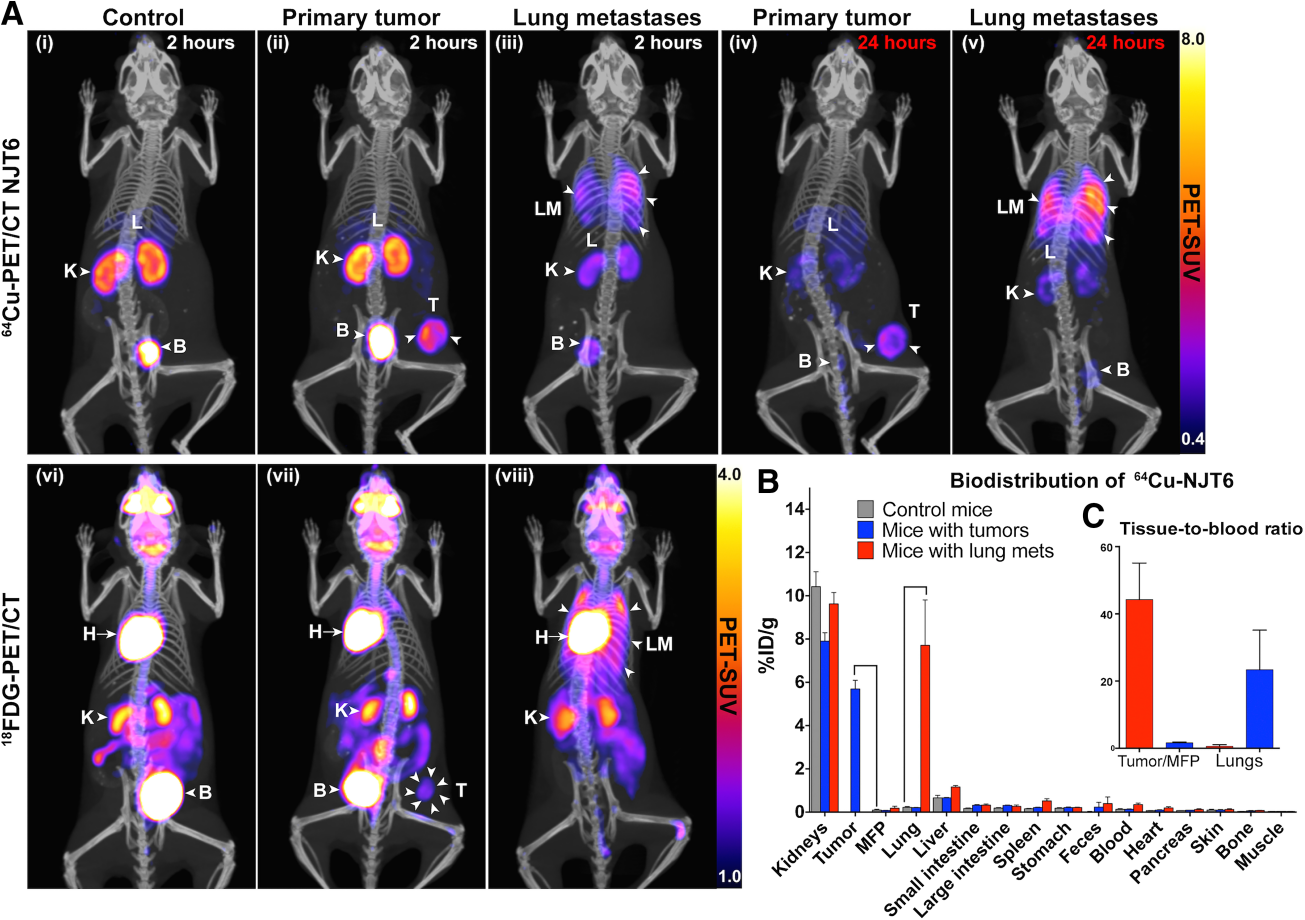
NJT6 detects tumors and lung metastases *in vivo* by immuno-PET/CT imaging with a high signal-to-noise ratio and low background. **A,** LM2 cells were injected in the MFP or via the tail vein of NSG mice to induce primary tumors or pulmonary metastases, respectively. Three weeks after tumor initiation, control and tumor-bearing mice were imaged with 64Cu-NJT6 immuno-PET/CT (top) or 18F-FDG PET/CT (bottom), Representative immuno-PET/CT images of maximum-intensity projections of mice imaged at 2 hours post-injection are shown. (i) NSG control mice; signals detected in kidney (K), liver (L), and bladder (B). (ii) Mouse bearing primary tumor; strong signals also detected in primary tumor (T). (iii) Mouse bearing pulmonary metastases; strong signals also detected in lung metastases (LM). (iv) Mouse bearing primary tumor imaged 24 hours after injection with labeled nanobodies; signals still detected in primary tumor. (v) Mouse bearing pulmonary metastases imaged 24 hours post-injection; signals still detected in lung metastases. (vi–viii) Representative images of control mice, mice with primary tumors, and mice with pulmonary metastases imaged with 18F-FDG PET/CT. H, heart. Images are representative of 6 to 7 mice with similar results. **B,***Ex vivo* biodistribution of 64Cu-NJT6, expressed as %ID/g in various organs. *N* = 3 for all groups. **C,** Inset, data in **B** expressed as tissue-to-blood ratios for tumors/MFPs and lungs.

The *in vivo* specificity of the ^64^Cu-labeled nanobodies was further tested by immuno-PET/CT imaging, a noninvasive imaging approach where antibody fragments are tagged with PET probes and injected into animals to assess antibody-target engagement. LM2-TGL-ZsGreen cells were injected into the MFPs of NSG mice for orthotopic tumor induction or via the lateral tail vein for induction of pulmonary metastases. For PET/CT imaging, control mice (no tumors), mice with mammary tumors, and mice with pulmonary metastases were injected intravenously with ^64^Cu-labeled nanobodies. Two hours after injection, the mice were imaged by PET imaging followed by CT for anatomic referencing. For all 3 nanobodies, in all mice, signals were observed in the kidneys and bladder; lower signals were also observed from the liver (Fig. 6A, top; Supplementary Fig. S6). These are the routes of clearance of the nanobody and of free copper-64. In the case of ^64^Cu-NJT6, in mice with primary tumors, in addition to the clearance organs (kidney, bladder, and liver), clear and bright signals were observed from the site of the primary tumor (Fig. 6A, **ii**, top). Similarly, in mice with pulmonary metastases, in addition to the signal from the clearance organs, clear signals were observed from the lungs (Fig. 6A, **iii**, top). In both models, the immuno-PET imaging showed low background and excellent clarity. Further investigation of the specificity of NJT6 for its presumed target (TNC) in the PET imaging, using for example competition by unlabeled nanobody (1) would be necessary for detailed characterization of TNC in tumors and metastases but the data presented here clearly support the selective binding of this nanobody to malignant tissues.

In comparison with ^64^Cu-NJT6, ^64^Cu-NJT3 and ^64^Cu-NJT4 also recognized both primary tumors and lung metastases, but exhibited more background signal than NJT6, most notably in the liver but also more broadly (Supplementary Fig. S6A and S6B). Compared with ^64^Cu-NJT6, the higher background in multiple organs including the kidneys, liver, and intestines observed for ^64^Cu-NJT3 and ^64^Cu-NJT4 nanobodies may have led to relatively slower blood-clearance or vice versa. Thus, in choosing nanobodies for use in imaging or targeting, it will be important to screen them for their background signals and speed of clearance from the circulation, which can vary among different nanobodies for a variety of reasons.

To test whether the nanobodies remain bound to the target site, the mice were also imaged 24 hours after ^64^Cu-nanobody injections (Fig. 6; Supplementary Fig. S6). In the case of ^64^Cu-NJT6, in mice bearing primary tumors, strong signals were still observed from the site of the tumor, albeit lower than observed at 2 hours (Fig. 6A, **iv**). In mice with pulmonary metastases, clear and strong signals were observed from the lungs (Fig. 6A, **v**). The signals from the kidneys and bladder were much lower at 24 hours in both models, suggesting that the nanobody had almost cleared from circulation and excretion pathways in these mice (Fig. 6A, **iv** and **v**). Signals at sites of primary tumors and lung metastases were also observed at 24 hours for mice imaged with ^64^Cu-NJT3 and ^64^Cu-NJT4 (Supplementary Fig. S6A and S6B).

For comparison, all mice imaged with immuno-PET/CT were also imaged with ^18^F-FDG PET/CT, the conventional imaging modality used in the clinic. ^18^F-FDG is taken up by metabolically active sites in the organism such as sites of tumors and metastases (35). In all mice tested, although signals were observed from the site of disease, the tumors and lung metastases were not detected as clearly as they were with the ^64^Cu-labeled nanobodies (Fig. 6A, **vii** and **viii**, bottom). Also, in addition to signals from the clearance organs, multiple additional ^18^F-FDG signals were observed from the heart, gut, harderian glands, and spleen, resulting in a higher background, lower signal-to-noise ratio, and lower selectivity. Similar ^18^F-FDG results were observed for mice also imaged with ^64^Cu-NJT3 and ^64^Cu-NJT4.

For organ biodistribution analysis, mice were euthanized after immuno-PET/CT imaging and multiple organs were resected (see Supplementary Methods). The biodistribution of ^64^Cu-NJT6 calculated as %ID/g is shown in Fig. 6B. While in most organs the values were comparable, the uptake of ^64^Cu-NJT6 was much higher for tumors (compared with MFPs of control mice) and lungs with metastases (compared with lungs of control mice). There was nearly 35-fold higher accumulation of the nanobody in the lungs of mice with lung metastases compared with control mice. Similarly, there was ∼80-fold higher accumulation of the nanobody in mammary tumors compared with the contralateral MFPs. The tissue-to-blood ratios for the tumors (44.26) and lung metastases (23.36) were remarkably high and markedly higher than controls (Fig. 6C).

The biodistribution analysis for ^64^Cu-NJT3 and ^64^Cu-NJT4 showed that the highest uptake of the Cu-labeled nanobodies was seen in the tumors (Supplementary Fig. S6C and S6D). The lungs of mice with pulmonary metastases had higher signals compared with control mice. However, multiple other organs such as liver, small intestine, spleen, and stomach had relatively higher uptake than that observed for the same organs during ^64^Cu-NJT6 imaging (Supplementary Fig. S6C and S6D compared with Fig. 6B). ^64^Cu-NJT3 and ^64^Cu-NJT4 also had lower tissue-to-blood ratios compared with ^64^Cu-NJT6 (Supplementary Fig. S6). These data show that ^64^Cu-NJT3 and ^64^Cu-NJT4 recognize tumors and metastases *in vivo*, albeit with lower signal-to-noise ratio and more background than ^64^Cu-NJT6.

IHC with biotin-labeled NJT6 on multiple mouse organs resected from NOD/SCID mice confirms that the nanobody binds specifically to the hTNC protein expressed in the ECM of the tumors and metastases derived from the human LM2 TNBC cells (Supplementary Table S3). We note here that compared with a cross-reactive control antibody that binds some normal murine tissues (Supplementary Table S3), the use of a nanobody that only recognizes human TNC, may have resulted in preferential apparent tumor selectivity in mouse xenografts and the selectivity for tumor tissues needs additional verification.

Together these data show that all three nanobodies bind to hTNC in the ECM of tumors and metastases *in vivo* with higher clarity and better signal-to-noise ratio than ^18^F-FDG-PET/CT imaging. The best among these, the ^64^Cu-labeled NJT6 emerged as the preferred candidate for noninvasive *in vivo* applications. Its remarkably high tumor-to-blood ratio, fast clearance, low background, excellent biodistribution properties and high specificity establish it as a promising tool for monitoring tumor progression and metastases. This also demonstrates its potential application as a carrier of therapeutic cargo.

## Discussion

To expand our understanding of the nature of the ECM in human metastases, in this paper, we characterized the ECM of 6 patient metastases from two different tumor types; TNBC metastases to liver and lung and colorectal cancer metastases to the liver (Fig. 1). Using LC-MS/MS, we identified 220 matrisome proteins in these samples and a signature of 67 matrisome proteins that were shared among all patient samples analyzed (Supplementary Table S1).

We explored the expression of the proteins in our signature across other matrisome datasets and report that a great majority of the ECM proteins identified in this 67-protein ECM signature are also abundant in the ECM of human tumors of the breast, pancreas, and colon (Fig. 2D). Many proteins were also reported to be significantly abundant in the ECM of metastases derived from ovarian tumors and colorectal cancer. (Fig. 2D). We further examined the mRNA expression of this 67-gene set in TCGA breast cancer datasets of primary breast tumors, normal breast, and breast cancer metastases. The purpose of the latter analysis was to explore the expression of the metastasis-associated signature at the gene level and assess whether there are shared features between the expression of these genes in primary tumors and metastases. PCAs of the expression of all matrisome genes and of the 67-gene signature showed that the metastases co-clustered with the primary tumor cluster, and this cluster was distinct from the normal tissues, indicating that the expression levels of matrisome genes were closer in primary tumors and metastases as compared with normal breast ECM. Our results highlight that similarities in the ECM of tumors and metastases are a common feature across breast cancer patients. Similar observations have been made for human colorectal cancer tumors and matched liver metastases, albeit from a limited set of patients (24). Our data could be enriched with deeper proteomics to further refine the metastasis-associated signature (see Materials and Methods). Collectively, our findings reflect the literature that the ECM or “matrisome” changes markedly during tumor progression and metastasis (26, 30, 31), suggesting common stromal responses during the process. This strengthens our hypothesis that the matrisome as a compartment of the TME provides an important target in cancer and that the shared features across primary tumors and metastases are promising targets for diagnostics monitoring and therapy.

In the context of both imaging and therapy, an optimal target is one whose expression is: (i) relatively abundant in diseased tissue relative to normal and (ii) relatively homogeneous in the disease site (primary tumors and metastases) and across patient samples. A protein whose expression is shared among cancer types is an even more attractive target. Comparison of our matrisome signature with previously published ECM signatures and with immuno-histochemical validation further narrowed the list of potential ECM targets that are not only shared between metastases and primary tumors across cancer types, but whose expression is restricted to diseased tissues. The role of some of these proteins such as TNC and POSTN is well established in metastases and approaches to target several are already underway such as POSTN, FN, TNC, and collagens (36). Various efforts have been made to target TNC for diagnosis and therapy (25, 37–40). Several anti-TNC agents are in clinical trials; however, no agents are currently approved for clinical use (41). Others like TGFBI are potential targets yet to be explored.

We have previously shown that ECM-specific nanobodies, such as FN-EIIIB-specific NJB2, can be excellent tools for *in vitro* and *in vivo* applications including noninvasive immuno-PET/CT imaging (1) as well as targeting potential therapeutics to tumors and importantly metastases (2). Nanobodies have various therapeutic applications (42) and can be readily generated, selected and engineered. They are monomeric, stable, bind targets with high affinity, have fast renal clearance and are a renewable resource (28, 43, 44). Immunizing alpacas with complex ECM preparations from patient metastases is one way to obtain nanobody libraries that contain binders to multiple proteins that could serve as shared therapeutic targets for both primary tumors and their metastases. The NJB2 nanobody was derived from an alpaca immunized with a cocktail of select purified ECM proteins and peptides. Immunizing with purified soluble recombinant proteins is the most common and preferred method used for generating immune Nb libraries (28, 45). In contrast, here we report libraries generated from insoluble and cross-linked complex mixtures of ECM derived directly from patient metastasis samples. Use of the insoluble, cross-linked ECM as immunogen may also act as a sort of pseudo-adjuvant and enhance the immunogenicity for particular ECM proteins. With a diversity of 10^7^ to 10^8^ these libraries present a large and diverse collection of binders to the disease ECM. Nanobodies derived from these libraries can be used as tools for the characterization and manipulation of the ECM, both *in vitro* and *in vivo*.

To demonstrate the potential of our approach, we chose TNC as an example target protein. TNC is known to be abundant in the ECM of primary tumors. It shows restricted expression in normal adult tissues (25) and, based on our proteomic analysis of human metastatic samples, it is one of the more abundant ECM proteins present in all 6 patients. It was detected in mammary tumors and also present in three of the four tumor ECM datasets (Fig. 2D). TNC is known to play a role in metastasis and is a key molecule in the metastatic niche (34, 46); and we report that a large percentage of human metastases from multiple origins and at different sites express TNC (Fig. 3). Although some of the metastasis biopsy samples in the TMA were negative for TNC expression, this may be attributable to heterogeneous expression of TNC across the tumor such that small biopsies would score negative. This may not be an issue for *in vivo* imaging and therapeutic approaches where the entire tumor or metastases are imaged or treated.

We isolated 3 high-affinity nanobodies with subpicomolar affinities for human TNC and showed that all were able to bind small metastases (including micrometastases; Figs. 4 and 5). We developed the nanobodies into PET radiotracers and evaluated them for noninvasive imaging using immuno-PET/CT, which showed higher clarity and better signal-to-noise ratios compared with conventional ^18^F-FDG-PET/CT imaging. Therefore, they can be used to deliver diagnostic and therapeutic agents to disease sites, which are otherwise hard to reach by conventional therapeutic approaches. While all three nanobodies readily and specifically reach sites of primary tumors and occult metastases, the best among these, NJT6, exhibited a very high tumor-to-blood ratio and desirable biodistribution properties (Fig. 6B). The divergent *in vivo* behaviors of these three nanobodies despite similar molecular weights and affinity to the target may be due to differences in nonspecific background or to several other factors that were not explored in this study but have been shown to impact the *in vivo* pharmacokinetics of antibodies and peptides such as charge heterogeneity, hydrophobicity, *in vivo* stability and modifications among others (47). These observations highlight the necessity of screening multiple nanobodies to a given target to find those best suited for *in vivo* applications.

Although TNC is known to play a role in breast cancer metastases, we have not yet investigated the role of these nanobodies as function-blocking antibodies such as by studying their impact on tumor progression and/or metastatic dissemination. This is a proof-of-principle study and the breadth of high affinity ECM binders contained within these libraries remains to be explored. One caveat of the current study is that the human-specific TNC nanobodies have been tested in a murine background. While TNC is known to show restricted expression in normal tissues (25), the inability to recognize murine TNC present in certain normal tissues, could contribute to the low background signals observed during PET/CT imaging. Understanding potential toxicities of anti-TNC agents is important. Therefore, isolation of human/mouse cross-reactive nanobodies would be an attractive approach for further pre-clinical testing and translation. Other antibody formats that are specific to certain domains (A1 and D) of TNC have been developed and tested for imaging and therapy (39, 40). However, the NJT6 nanobody reported here shows the highest affinity and excellent biodistribution profiles with high tumor-to-blood ratios within 2 hours of injection; desirable properties in the context of both imaging and therapy.

Nanobodies in these libraries can have many other potential uses. Depending on the intended outcome, the ECM can be targeted in multiple ways, such as interfering with its synthesis, remodeling, assembly, cross-linking and by blocking growth factor-ECM interactions. The ECM can be used as an anchor to deliver therapeutic and diagnostic agents or by specifically targeting ECM proteins that are causal to tumorigenesis and metastases (via function-blocking antibodies; refs. 36, 48–50). Our libraries provide many potential binders that can be explored for these diverse applications. Importantly, these nanobodies have sortase tags that allow them to be readily engineered to deliver imaging and therapeutic cargo.

In conclusion, this approach of screening for ECM proteins or epitopes that are strongly and selectively expressed in tumors and/or metastases, coupled with generation of libraries of (monoclonal) nanobodies to metastatic ECM offers the potential to identify and exploit additional tumor biomarkers as targets for imaging and for systemic delivery of various agents selectively targeted to tumor sites.

## Supplementary Material

Table S1 Supplementary Table S1

Table S2 Supplementary Table S2

Table S3 Supplementary Table S3

Supplementary Data Supplementary methods and materials.

## References

[1] Jailkhani N , Ingram JR, Rashidian M, Rickelt S, Tian C, Mak H, . Noninvasive imaging of tumor progression, metastasis, and fibrosis using a nanobody targeting the extracellular matrix. Proc Natl Acad Sci USA 2019;116:14181–90. 31068469 10.1073/pnas.1817442116 PMC6628802

[2] Xie YJ , Dougan M, Jailkhani N, Ingram J, Fang T, Kummer L, . Nanobody-based CAR T cells that target the tumor microenvironment inhibit the growth of solid tumors in immunocompetent mice. Proc Natl Acad Sci USA 2019;116:7624–31. 30936321 10.1073/pnas.1817147116 PMC6475367

[3] Lim SB , Tan SJ, Lim WT, Lim CT. An extracellular matrix-related prognostic and predictive indicator for early-stage non–small cell lung cancer. Nat Commun 2017;8:1734. 29170406 10.1038/s41467-017-01430-6 PMC5700969

[4] Pearce OMT , Delaine-Smith RM, Maniati E, Nichols S, Wang J, Bohm S, . Deconstruction of a metastatic tumor microenvironment reveals a common matrix response in human cancers. Cancer Discov 2018;8:304–19. 29196464 10.1158/2159-8290.CD-17-0284 PMC5837004

[5] Socovich AM , Naba A. The cancer matrisome: from comprehensive characterization to biomarker discovery. Semin Cell Dev Biol 2019;89:157–66. 29964200 10.1016/j.semcdb.2018.06.005

[6] Yuzhalin AE , Urbonas T, Silva MA, Muschel RJ, Gordon-Weeks AN. A core matrisome gene signature predicts cancer outcome. Br J Cancer 2018;118:435–40. 29360819 10.1038/bjc.2017.458 PMC5808042

[7] Schedin P , Keely PJ. Mammary gland ECM remodeling, stiffness, and mechanosignaling in normal development and tumor progression. Cold Spring Harb Perspect Biol 2011;3:a003228. 20980442 10.1101/cshperspect.a003228 PMC3003460

[8] Hynes RO , Naba A. Overview of the matrisome–an inventory of extracellular matrix constituents and functions. Cold Spring Harb Perspect Biol 2012;4:a004903. 21937732 10.1101/cshperspect.a004903 PMC3249625

[9] Charras G , Sahai E. Physical influences of the extracellular environment on cell migration. Nat Rev Mol Cell Biol 2014;15:813–24. 25355506 10.1038/nrm3897

[10] Rozario T , DeSimone DW. The extracellular matrix in development and morphogenesis: a dynamic view. Dev Biol 2010;341:126–40. 19854168 10.1016/j.ydbio.2009.10.026 PMC2854274

[11] Hynes RO . The extracellular matrix: not just pretty fibrils. Science 2009;326:1216–9. 19965464 10.1126/science.1176009 PMC3536535

[12] Bonnans C , Chou J, Werb Z. Remodelling the extracellular matrix in development and disease. Nat Rev Mol Cell Biol 2014;15:786–801. 25415508 10.1038/nrm3904 PMC4316204

[13] Iozzo RV , Gubbiotti MA. Extracellular matrix: the driving force of mammalian diseases. Matrix Biol 2018;71–72:1–9. 10.1016/j.matbio.2018.03.023 PMC6146050 29625183

[14] Lu P , Takai K, Weaver VM, Werb Z. Extracellular matrix degradation and remodeling in development and disease. Cold Spring Harb Perspect Biol 2011;3:a005058. 21917992 10.1101/cshperspect.a005058 PMC3225943

[15] Cox TR , Erler JT. Remodeling and homeostasis of the extracellular matrix: implications for fibrotic diseases and cancer. Dis Model Mech 2011;4:165–78. 21324931 10.1242/dmm.004077 PMC3046088

[16] Matsubayashi Y , Sanchez-Sanchez BJ, Marcotti S, Serna-Morales E, Dragu A, Diaz-de-la-Loza MD, . Rapid homeostatic turnover of embryonic ECM during tissue morphogenesis. Dev Cell 2020;54:33–42. 32585131 10.1016/j.devcel.2020.06.005 PMC7332994

[17] Barker HE , Paget JT, Khan AA, Harrington KJ. The tumor microenvironment after radiotherapy: mechanisms of resistance and recurrence. Nat Rev Cancer 2015;15:409–25. 26105538 10.1038/nrc3958 PMC4896389

[18] Henke E , Nandigama R, Ergun S. Extracellular matrix in the tumor microenvironment and its impact on cancer therapy. Front Mol Biosci 2019;6:160. 32118030 10.3389/fmolb.2019.00160 PMC7025524

[19] Fatherree JP , Guarin JR, McGinn RA, Naber SP, Oudin MJ. Chemotherapy-induced collagen IV drives cancer cell motility through activation of Src and focal adhesion kinase. Cancer Res 2022;82:2031–44. 35260882 10.1158/0008-5472.CAN-21-1823 PMC9381104

[20] Shao X , Taha IN, Clauser KR, Gao YT, Naba A. MatrisomeDB: the ECM-protein knowledge database. Nucleic Acids Res 2020;48:D1136–D44. 31586405 10.1093/nar/gkz849 PMC6943062

[21] Naba A , Clauser KR, Hoersch S, Liu H, Carr SA, Hynes RO. The matrisome: in silico definition and *in vivo* characterization by proteomics of normal and tumor extracellular matrices. Mol Cell Proteomics 2012;11:M111 014647. 10.1074/mcp.M111.014647 PMC3322572 22159717

[22] Hebert JD , Myers SA, Naba A, Abbruzzese G, Lamar JM, Carr SA, . Proteomic profiling of the ECM of xenograft breast cancer metastases in different organs reveals distinct metastatic niches. Cancer Res 2020;80:1475–85. 32019869 10.1158/0008-5472.CAN-19-2961 PMC7127975

[23] Mayorca-Guiliani AE , Madsen CD, Cox TR, Horton ER, Venning FA, Erler JT. ISDoT: in situ decellularization of tissues for high-resolution imaging and proteomic analysis of native extracellular matrix. Nat Med 2017;23:890–8. 28604702 10.1038/nm.4352

[24] Naba A , Clauser KR, Whittaker CA, Carr SA, Tanabe KK, Hynes RO. Extracellular matrix signatures of human primary metastatic colon cancers and their metastases to liver. BMC Cancer 2014;14:518. 25037231 10.1186/1471-2407-14-518 PMC4223627

[25] Midwood KS , Chiquet M, Tucker RP, Orend G. Tenascin-C at a glance. J Cell Sci 2016;129:4321–7. 27875272 10.1242/jcs.190546

[26] Naba A , Clauser KR, Lamar JM, Carr SA, Hynes RO. Extracellular matrix signatures of human mammary carcinoma identify novel metastasis promoters. Elife 2014;3:e01308. 24618895 10.7554/eLife.01308 PMC3944437

[27] Ingram JR , Knockenhauer KE, Markus BM, Mandelbaum J, Ramek A, Shan Y, . Allosteric activation of apicomplexan calcium-dependent protein kinases. Proc Natl Acad Sci USA 2015;112:E4975–84. 26305940 10.1073/pnas.1505914112 PMC4568647

[28] Muyldermans S . A guide to: generation and design of nanobodies. FEBS J 2021;288:2084–102. 32780549 10.1111/febs.15515 PMC8048825

[29] Guimaraes CP , Witte MD, Theile CS, Bozkurt G, Kundrat L, Blom AE, . Site-specific C-terminal and internal loop labeling of proteins using sortase-mediated reactions. Nat Protoc 2013;8:1787–99. 23989673 10.1038/nprot.2013.101 PMC3943461

[30] Tian C , Clauser KR, Ohlund D, Rickelt S, Huang Y, Gupta M, . Proteomic analyses of ECM during pancreatic ductal adenocarcinoma progression reveal different contributions by tumor and stromal cells. Proc Natl Acad Sci USA 2019;116:19609–18. 31484774 10.1073/pnas.1908626116 PMC6765243

[31] Gocheva V , Naba A, Bhutkar A, Guardia T, Miller KM, Li CM, . Quantitative proteomics identify tenascin-C as a promoter of lung cancer progression and contributor to a signature prognostic of patient survival. Proc Natl Acad Sci USA 2017;114:E5625–E34. 28652369 10.1073/pnas.1707054114 PMC5514763

[32] Rickelt S , Hynes RO. Antibodies and methods for immunohistochemistry of extracellular matrix proteins. Matrix Biol 2018;71–72:10–27. 10.1016/j.matbio.2018.04.011 29730502

[33] Lowy CM , Oskarsson T. Tenascin-C in metastasis: A view from the invasive front. Cell Adh Migr 2015;9:112–24. 25738825 10.1080/19336918.2015.1008331 PMC4422797

[34] Oskarsson T , Acharyya S, Zhang XH, Vanharanta S, Tavazoie SF, Morris PG, . Breast cancer cells produce tenascin-C as a metastatic niche component to colonize the lungs. Nat Med 2011;17:867–74. 21706029 10.1038/nm.2379 PMC4020577

[35] Zhu A , Lee D, Shim H. Metabolic positron emission tomography imaging in cancer detection and therapy response. Semin Oncol 2011;38:55–69. 21362516 10.1053/j.seminoncol.2010.11.012 PMC3075495

[36] Venning FA , Wullkopf L, Erler JT. Targeting ECM disrupts cancer progression. Front Oncol 2015;5:224. 26539408 10.3389/fonc.2015.00224 PMC4611145

[37] Hicke BJ , Stephens AW, Gould T, Chang YF, Lynott CK, Heil J, . Tumor targeting by an aptamer. J Nucl Med 2006;47:668–78. 16595502

[38] Lingasamy P , Tobi A, Kurm K, Kopanchuk S, Sudakov A, Salumae M, . Tumor-penetrating peptide for systemic targeting of tenascin-C. Sci Rep 2020;10:5809. 32242067 10.1038/s41598-020-62760-y PMC7118115

[39] Brack SS , Silacci M, Birchler M, Neri D. Tumor-targeting properties of novel antibodies specific to the large isoform of tenascin-C. Clin Cancer Res 2006;12:3200–8. 16707621 10.1158/1078-0432.CCR-05-2804

[40] Nadal L , Corbellari R, Villa A, Weiss T, Weller M, Neri D, . Novel human monoclonal antibodies specific to the alternatively spliced domain D of tenascin-C efficiently target tumors *in vivo*. MAbs 2020;12:1836713. 33136526 10.1080/19420862.2020.1836713 PMC7646483

[41] Spenle C , Saupe F, Midwood K, Burckel H, Noel G, Orend G. Tenascin-C: Exploitation and collateral damage in cancer management. Cell Adh Migr 2015;9:141–53. 25569113 10.1080/19336918.2014.1000074 PMC4422814

[42] Steeland S , Vandenbroucke RE, Libert C. Nanobodies as therapeutics: big opportunities for small antibodies. Drug Discov Today 2016;21:1076–113. 27080147 10.1016/j.drudis.2016.04.003

[43] Ingram JR , Schmidt FI, Ploegh HL. Exploiting nanobodies' singular traits. Annu Rev Immunol 2018;36:695–715. 29490163 10.1146/annurev-immunol-042617-053327

[44] Muyldermans S . Nanobodies: natural single-domain antibodies. Annu Rev Biochem 2013;82:775–97. 23495938 10.1146/annurev-biochem-063011-092449

[45] Pardon E , Laeremans T, Triest S, Rasmussen SG, Wohlkonig A, Ruf A, . A general protocol for the generation of nanobodies for structural biology. Nat Protoc 2014;9:674–93. 24577359 10.1038/nprot.2014.039 PMC4297639

[46] Oskarsson T , Massague J. Extracellular matrix players in metastatic niches. EMBO J 2012;31:254–6. 22179697 10.1038/emboj.2011.469 PMC3261570

[47] Datta-Mannan A . Mechanisms influencing the pharmacokinetics and disposition of monoclonal antibodies and peptides. Drug Metab Dispos 2019;47:1100–10. 31043438 10.1124/dmd.119.086488

[48] Mansurov A , Ishihara J, Hosseinchi P, Potin L, Marchell TM, Ishihara A, . Collagen-binding IL-12 enhances tumor inflammation and drives the complete remission of established immunologically cold mouse tumors. Nat Biomed Eng 2020;4:531–43. 32284554 10.1038/s41551-020-0549-2 PMC11095084

[49] Weiss T , Puca E, Silginer M, Hemmerle T, Pazahr S, Bink A, . Immunocytokines are a promising immunotherapeutic approach against glioblastoma. Sci Transl Med 2020;12:eabb2311. 33028706 10.1126/scitranslmed.abb2311

[50] Momin N , Palmeri JR, Lutz EA, Jailkhani N, Mak H, Tabet A, . Maximizing response to intratumoral immunotherapy in mice by tuning local retention. Nat Commun 2022;13:109. 35013154 10.1038/s41467-021-27390-6 PMC8748612

