# Supplementary material for: Proteomic Profiling of Extracellular Matrix Components from Patient Metastases Identifies Consistently Elevated Proteins for Developing Nanobodies That Target Primary Tumors and Metastases
Source: Cancer Res. 2023 Apr 26;83(12):2052–65. doi: 10.1158/0008-5472.CAN-22-1532 (PMC10267678; doi:10.1158/0008-5472.CAN-22-1532)
Supplement: Supplementary Data — Supplementary methods and materials. [file can-22-1532_supplementary_data_suppsmm.pdf]

<u>Supplementary information for Jailkhani *et al.*, Cancer Research</u>

This PDF file includes:

Supplementary figures and legends (Fig S1 to S6)

Supplementary materials and methods

Supplementary table legends

Supplementary references

Other supplementary materials for this manuscript include the following:

Supplemental table 1-3

<u>Supplementary Figure and Legends</u>

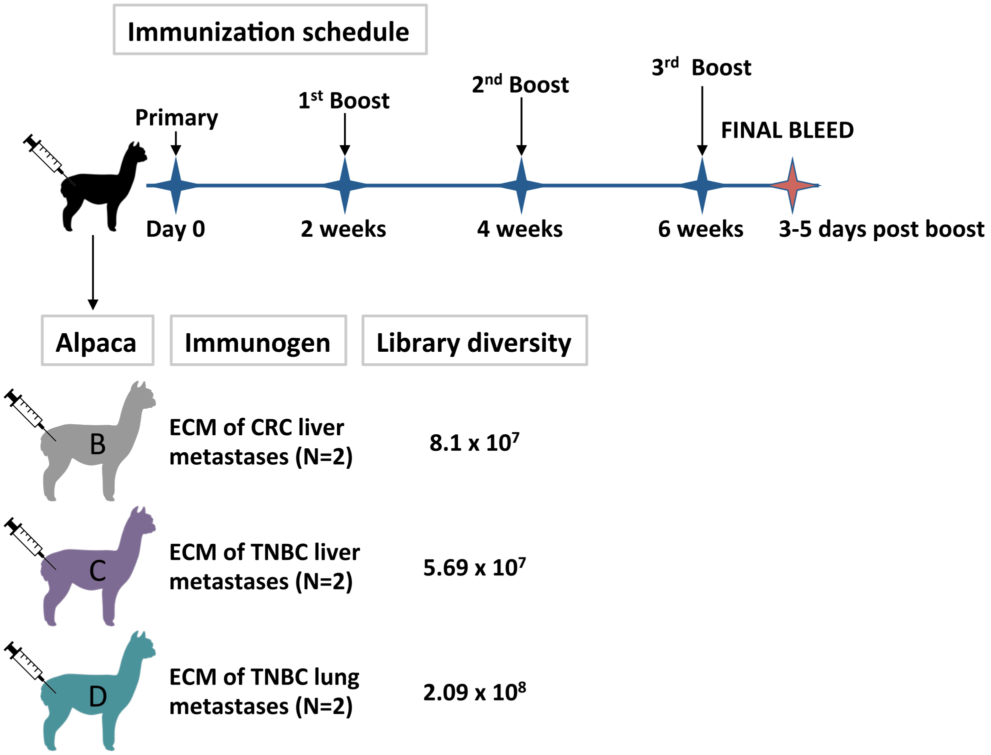

Supplementary Fig 1
Immunization schedule for nanobody generation (top). Details of immunogens injected into Alpacas B, C and D and diversities (in colony-forming unit or CFUs) of the corresponding nanobody libraries derived from these alpacas are indicated.

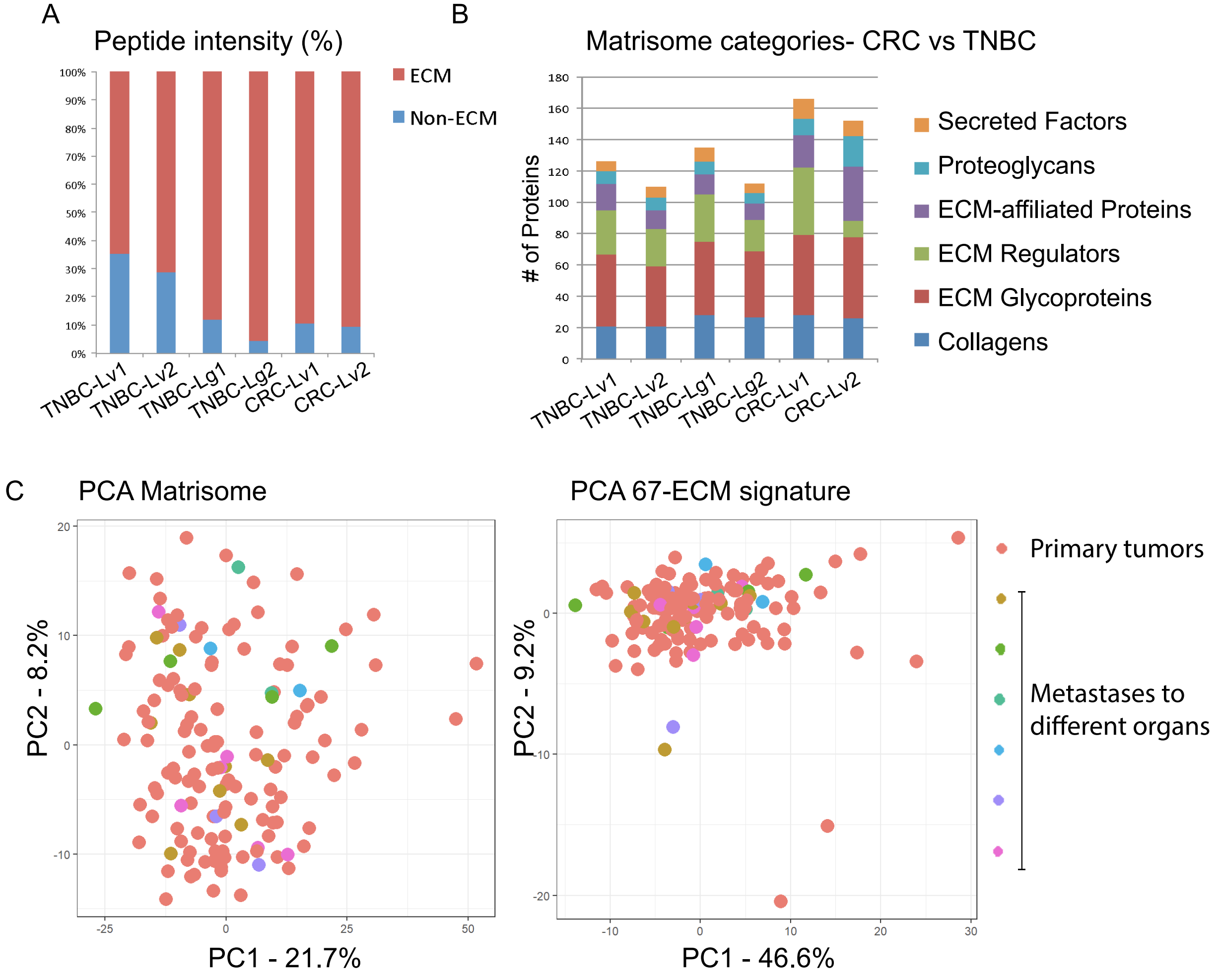

Supplementary Fig S2

(**A**) Distribution of peptides from non-matrisome (blue) and matrisome (red) proteins identified by proteomics across different patient metastasis samples of TNBC liver metastases (TNBC Lv1 and Lv2), TNBC lung metastases (TNC Lg1 and Lg2) and colorectal cancer liver metastases (CRC Lv1 and Lv2). Bar graph shows comparisons of total abundance from precursor-ion peak areas for all peptides identified in each sample. Data shown are the sum of duplicate samples from each metastasis. (**B**) Comparison of the number of "core matrisome" (Collagens, ECM glycoproteins and proteoglycans) and "matrisome-associated" proteins (ECM regulators, secreted factors and affiliated proteins) identified across patient metastasis samples. (**C**) PCA plots showing expression of matrisome genes and 67-ECM signature from RNA-seq data derived from the metastatic breast cancer project dataset. Both plots show a co-clustering of the metastases from different sites (multiple colored) with the primary tumor cluster (red).

A

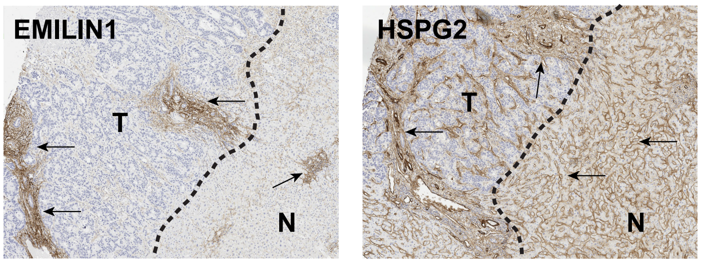

B

	Primary site	Both positive biopsy	Single positive biopsy	Metastatic site				
1	Bladder	2	1	Pelvic cavity LN (3)	Back (2)			
2	Breast	1	2	Armpit LN (3)	Occipital lobe			
3	Cervix	3		Colon (2)	Pelvic LN (4)			
4	Colon	2		Mesentery LN (4)				
5	Duodenum	2		Omentum LN (2)	Mesentry LN (2)			
6	Endometrium	1	2	Abdominal cavity (3)	Vaginal wall			
7	Kidney	2		Renal hilum LN (2)	Adrenal gland (2)			
8	Larynx	3	1	Neck (3)	Neck LN (4)			
9	Liver	1		Colon (2)				
10	Lung	6	1	Right femur (1)	Lymph node (10)	Left frontal lobe (2)		
11	Oesophagus	3		Around oesophagus (4)	Mediastinum (2)			
12	Ovary	2	3	Appendix (2)	Cervix	Omentum	Bladder (2)	Peritonium
13	Pancreas	5		Omentum (2)	Liver (2)	Omentum LN (6)		
14	Penis	2		Right groin (2)	Right groin LN (2)			
15	Prostate	1		Prostate LN (2)				
16	Rectum	3	1	Liver (7)				
17	Skin	2		Armpit LN (4)				
18	Small intestine	1		Mesentery LN (2)				
19	Stomach	3	1	Abdominal wall (2)	Omentum (3)	Cardia LN (2)		
20	Thyroid	4	1	Neck LN (9)				

Supplementary Fig S3

(**A**) Immunohistochemistry on a CRC liver metastasis sample shows that expression of EMILIN1 and HSPG2 is not restricted to the metastatic lesion ("T") but is also present in the adjacent normal liver tissue ("N"). Arrows point to regions of positive staining. (**B**) Summary table describing the primary sites/organs and the corresponding metastatic sites of biopsies that were positive for expression of stromal TNC. Number of patients with "both positive biopsies" and those with a "single positive biopsy" are also indicated. The numbers of biopsies positive at each metastatic site are indicated in the parentheses. The sites marked in blue are metastases present in the lymph nodes (LN) at the indicated site.

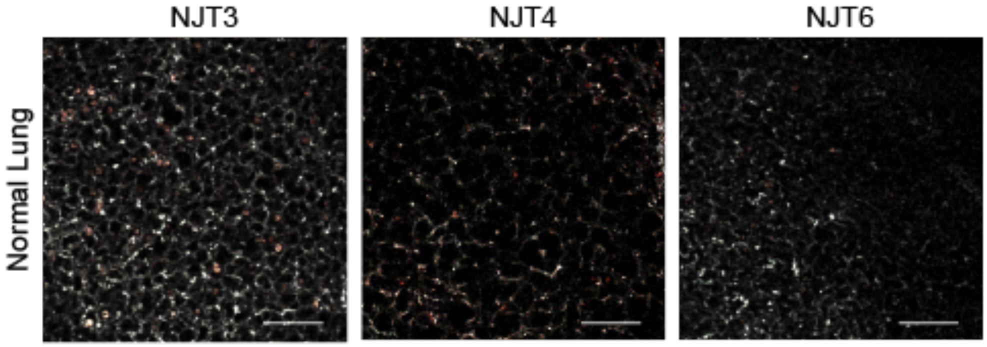

Supplementary Fig S4

α-TNC nanobodies did not bind to the ECM in normal lungs. Representative images are shown as an overlay of two different channels, grey (SHG, second harmonic generation) and red (labeled nanobody). No nanobody binding was observed in these normal lung tissues. (Scale bars, 100 um).

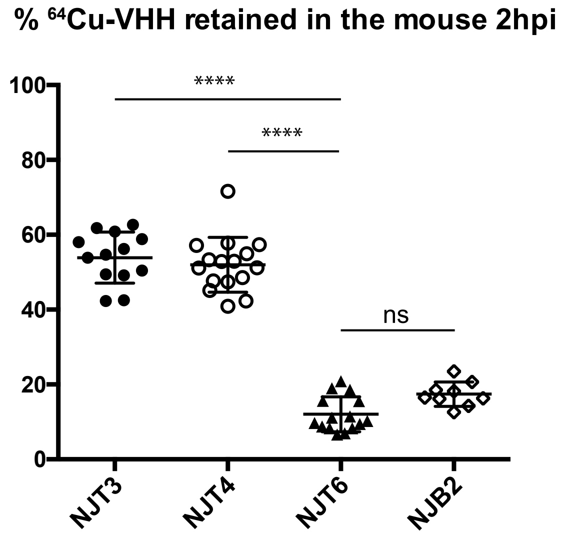

Supplementary Fig S5

Decay-corrected values of the activity injected into the mouse at T_0 (right after tail vein injection) and T_2 (2 hours after injection) were used to calculate the percentage of activity/nanobody retained in the mouse for each nanobody tested; NJT3/4/6 are anti-TNC and NJB2 is anti-EIIIB. Data were analyzed using one-way ANOVA, followed by post-hoc Tukey's multiple comparison tests. Each dot/symbol represents one mouse.

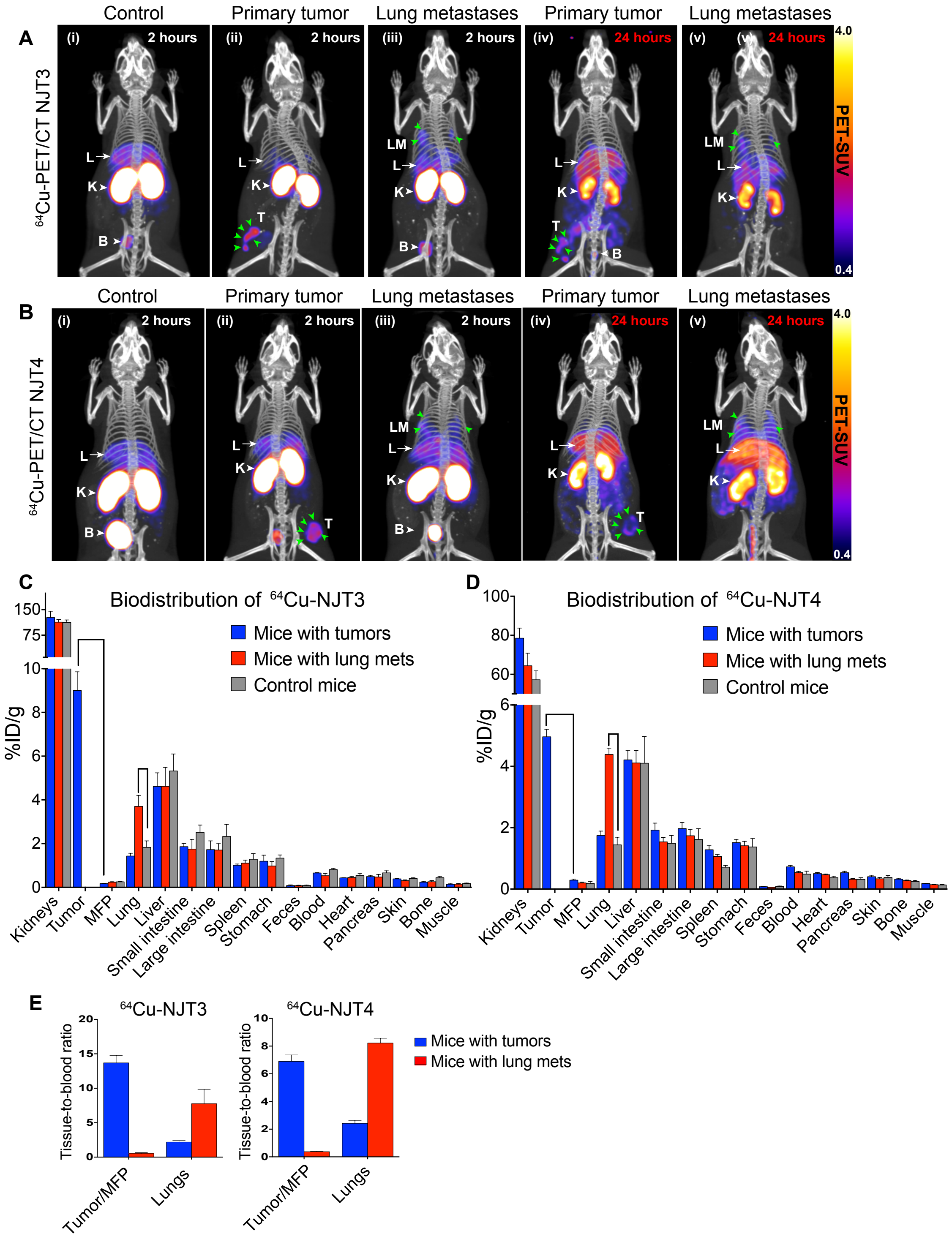

Supplementary Fig S6. NJT3 and NJT4 detect tumors and lung metastases albeit with lower signal-to-noise ratios and higher background.

(**A & B**) Representative immuno-PET/CT images for ^{64}Cu-NJT3 (A) and ^{64}Cu-NJT4 (**B**) at 2 and 24 hours after injection of radiolabelled nanobodies in (i) a control mouse, (ii) a mouse with primary tumor and (iii) a mouse with lung metastases. Signals were observed in the tumor ("T"), lung metastases ("LM"), kidneys ("K"), bladder ("B") and liver ("L"). For mice with tumors and lung metastases, images are representative of 6-7 mice with similar results. For controls, images are representative of 3-4 mice with similar results. (**C and D**). Ex vivo biodistribution of ^{64}Cu-NJT3 (C)

and ^{64}Cu-NJT4 (D), expressed as %ID/g in various organs in control mice, mice with tumors and lung metastases. For ^{64}Cu-NJT3, ~54 fold higher uptake was seen in the primary tumors (compared to contralateral mammary fat pads) and ~2.25 fold higher uptake in lung metastases (compared to normal lungs). For ^{64}Cu-NJT4, ~18 fold higher uptake was seen in the primary tumors (compared to contralateral mammary fat pads) and ~3 fold higher uptake in lung metastases (compared to normal lungs). For biodistribution analysis, N=3 for all groups. (**E**) Tissue-to-blood ratio of ^{64}Cu-NJT3 (left panel) and ^{64}Cu-NJT4 (right panel) in tumors, mammary fat pads (MFP) and lungs in mice with primary tumors and mice with lung metastases.

Supplementary materials and methods:

Sample preparation and alpaca immunization

To prepare the ECM-enriched pellets for immunization; the pellets from two different patients for each sample type were pooled. For the library derived from CRC liver metastases, ECM was enriched from 150 mg of metastatic tissue from each patient and the pellets from 2 different patients were pooled. For the libraries derived from TNBC liver and lung metastases, ECM was enriched from ~100 mg of metastatic tissue and the pellets from 2 different patients were pooled for immunization. These pooled pellets were then broken into smaller pieces by pipetting and vortexing in 500 ul of chilled PBS. The pellets were sonicated in a water bath sonicator on ice for 1 hour and further dissociated by passing through a 20G syringe needle. The samples were treated with UV light for 2 hours and divided into 4 equal doses and stored at -80°C. Before immunization, the samples were mixed with alum at a 1:1 volume/volume ratio. Three separate alpacas (B, C and D) were immunized using a protocol approved by UMass, Amherst and MIT IACUCs. Alpaca B was immunized with ECM enriched from liver metastases of two CRC patients, Alpaca C was immunized with ECM enriched from liver metastases of two TNBC patients and Alpaca D was immunized with ECM enriched from lung metastases of two TNBC patients. Following a primary immunization and three booster injections, RNA was extracted from peripheral blood lymphocytes and used to generate phage-display libraries using previously described methods (1,2).

NanoLC-MS/MS analysis

Each of the ECM extracts was reconstituted in 140-210 µl of 3% acetonitrile / 0.1% TFA and 1-3 µl (~1µg total peptide, measured by Nanodrop) analyzed by LC-MS/MS on a Q Exactive Plus mass spectrometer (Thermo Fisher Scientific, Waltham, MA) equipped with a nanoflow ionization source (James A. Hill Instrument Services, Arlington, MA) and coupled to an EASYnLC 1000 UHPLC system (Proxeon, Thermo Fisher Scientific). Chromatography was performed on a 75 um ID picofrit column (New Objective, Woburn, MA) packed in-house with ReprosilPur C18 AQ 1.9 um beads (Dr. Maisch, GmbH, Entringen, Germany) to a length of 20 cm. The column was heated to 50°C using a column heater sleeve (Phoenix-ST) to prevent overpressuring during UHPLC separation. The LC system, column, and platinum wire to deliver electrospray source voltage were connected via a stainless-steel cross (360µm, IDEX Health & Science, UH906x). The mobile-phase flow rate was 200nL/min and comprised of 3% acetonitrile/0.1% formic acid (Solvent A) and 90% acetonitrile / 0.1% formic acid (Solvent B). A 126-minute LCMS/MS method followed a 10-min column-equilibration procedure and a ~6-min sample-loading procedure for a 1-3 uL injection. The elution portion of the LC gradient was 2-5% solvent B in 2 min, 5-30% in 100 min, 30-60% in 10 min, 60-90% in 2 min, and held at 90% solvent B for 5min to yield ~15 sec peak widths. Data-dependent LC-MS/MS spectra were acquired in ~2 sec cycles; each cycle was of the following form: one full orbitrap MS scan at 70,000 resolution followed by 10 HCD MS/MS scans in the orbitrap at 17,500 resolution using an isolation width of 1.6 m/z with an offset of 0.3 m/z. Dynamic exclusion was enabled with a mass width of +/- 20 ppm, a repeat count of 1, and an exclusion duration of 30 sec. Charge-state screening was enabled to prevent triggering of MS/MS on precursor ions with unassigned charge or a charge state of 1. Monoisotopic precursor selection was used with peptide match set to preferred, and exclude isotopes on. For HCD MS/MS scans the normalized collision energy was 27, AGC target 5e4 ions, and max ion time 50 msec.

Protein/peptide identification

All LC-MS/MS data were interpreted using the Spectrum Mill (SM) software package v6.0 pre-release (proteomics.broadinstitute.org). Similar MS/MS spectra acquired on the same precursor m/z within +/- 40 sec were merged. MS/MS spectra were excluded from searching if they failed the quality filter by

not having a sequence tag length > 0 (i.e., minimum of two masses separated by the in-chain mass of an amino acid), did not have a precursor MH+ in the range of 600-6000, or a precursor charge > 5. MS/MS spectra were searched against a UniProt human sequence database containing reference proteome sequences (including isoforms and excluding fragments - 58,929 entries) downloaded from the UniProt web site on October 17, 2014, with a set of 150 common laboratory contaminant proteins appended. Search parameters included: ESI-QEXACTIVE-HCD-v2 scoring, parent and fragment mass tolerance of 20ppm, 40% minimum matched peak intensity, trypsin allow P enzyme specificity with up to 4 missed cleavages, and calculate reversed database scores enabled. Fixed modifications included carbamidomethylation at cysteine, while peptide N-termini were allowed to be modified either by carbamylation from urea, carbamidomethylation from iodoacetamide, or unmodified. Allowed variable modifications were acetylation of protein N-termini, oxidized methionine, deamidation of asparagine, pyro-glutamic acid at peptide N-terminal glutamine, pyro-carbamidomethylation at peptide N-terminal cysteine, and hydroxylation of proline with a precursor MH+ shift range of -18 to 97 Da. Hydroxyproline was only observed in the proteins known to have it (collagens and proteins containing collagen domains, emilins, etc.) and only within the expected GXPG sequence motifs.

Peptide spectrum matches (PSMs) were filtered with the SM autovalidation module to apply target-decoy-based false-discovery rate (FDR) thresholding via a two-step auto-threshold strategy at the peptide and protein levels to achieve a final peptide level FDR of 1% for each tumor type. First, peptide autovalidation was applied using an auto-thresholds strategy with a minimum sequence length of 7. For precursor charges 2-4: <1.6% FDR, automatic variable range precursor mass error filtering was applied to each LC-MS/MS run. For the less common precursor charge 5, <0.8% FDR was applied across both LC-MS/MS replicates together. Second, protein polishing autovalidation was applied with minimum protein group score: 15, and maximum protein group FDR: 0%, to eliminate low scoring PSM's from proteins identified by a single peptide from a single patient, so-called one-hit wonders. Proteins were considered quantifiable in a sample if they were represented by at least two peptides in one of the two biological replicates. For the intra-patient heterogeneity analysis, a protein was considered shared if it was detected in both replicates by at least one peptide.

Label-free Relative Protein Quantitation

Using the SM protein/peptide summary module identified proteins were combined into the same protein group if they shared a peptide with sequence length greater than 8. A protein group could be expanded into subgroups (isoforms or family members) when distinct peptides were present which uniquely represent a subset of the proteins in a group. Our *in silico* matrisome lists obtained from http://matrisome.org (3) were then used to categorize all of the identified protein groups as being ECM-derived or not. Protein groups and subgroups were quantified label-free using the abundance of each protein calculated as the sum of precursor ion chromatographic peak areas in MS1 spectra for all PSMs contributing to each protein group or subgroup. For simplicity, the quantitation presented in Figures 2A-B, 3A, S2A-B and Tables S1, S2 was with unexpanded protein groups. For table S2, the abundance values calculated are the mean/average of the two biological replicates for each sample.

The peak area for the extracted ion chromatogram of each precursor ion subjected to MS/MS was calculated automatically by the Spectrum Mill software in the intervening high-resolution MS1 scans of the LC-MS/MS runs using narrow windows around each individual member of the isotope cluster. Peak widths in both the time and *m*/*z* domains were dynamically determined based on MS scan resolution, precursor charge and *m*/*z,* subject to quality metrics on the relative distribution of the peaks in the isotope cluster vs theoretical. Although the determined protein abundances are generally reliable to within a factor of ~2 of the actual abundance, several experimental factors contribute to variability in the determined abundance for a protein. These factors include incomplete digestion of the protein;

widely varying response of individual peptides due to inherent variability in ionization efficiency as well as interference/suppression by other components eluting at the same time as the peptide of interest, differences in instrument sensitivity over the mass range analyzed, and sampling of the chromatographic peak between MS/MS scans.

Principal Component Analysis (PCA)
Count data for TCGA-BRCA samples were retrieved from the National Cancer Institute Genomic Data Commons Portal. Data for 1010 Matrisome genes or 67 GRC genes were extracted and principle component analysis was performed using the varianceStabilizingTransformation and plotPCA functions of DESeq2 (version 1.32.0) (4) in R version 4.1.0. The results shown here are in whole or part based upon data generated by the TCGA Research Network: https://www.cancer.gov/tcga. RNASeqV2 normalized data was obtained for the Metastatic Breast Cancer project from the cBioPortal interface (https://www.cbioportal.org/study/summary?id=brca_mbcproject_wagle_2017) and principle component analysis was done using prcomp and R version 4.1.0

Nanobody expression and purification
Nanobody expression and purification was done as previously described (1,2). Briefly, for periplasmic bacterial expression, nanobodies were expressed from the pHEN6 periplasmic expression vector in WK6 *E.coli* strain. The nanobody protein containing the LPETG sortase motif and His6-tag was purified from periplasmic extracts using Ni-NTA affinity purification (Qiagen) and size-exclusion chromatography with a Superdex 75 16/600 column; (GE Healthcare). Peak fractions were pooled and concentrated using Amicon 10-kDa molecular weight cut-off filtration unit (EMD Millipore) and stored at -80°C.

Sortase tagging
Sortase tagging was done as detailed previously (5). A pentamutant variant of *S. aureus* Sortase A was used to label the nanobodies by incubating 100-150 uM purified nanobody with 500uM nucleophile (GGGK-Biotin/GGGC-Texas Red/GGGC-Alexa 647/GGGC-NOTA) and 5 uM sortase in 50 mM Tris·HCl, pH 7.5, 150 mM NaCl, 10 mM CaCl2 for 2-6 h at 4 °C. Unreacted sortase and nanobody were removed by passing the reaction over Ni-NTA beads (Qiagen). The unbound fraction containing the tagged nanobody was concentrated, and excess nucleophile was removed with an Amicon 3,000-kDa MWCO filter.

Immunohistochemistry
IHC was performed as described previously (6). The following antibodies were used; anti-TNC (rabbit monoclonal, Abcam, #ab108930, 1:500), anti-HSPG2 (rabbit polyclonal, Boster, #PB9277, 1:1000), anti-POSTN (rabbit polyclonal, Abcam, #ab14041, 1:1000), anti-TGFBI (rabbit polyclonal, SigmaAldrich, #HPA017019, 1:500), anti-EMILIN1 (rabbit polyclonal, SigmaAldrich, #HPA002822, 1:500). IHC with biotinylated nanobodies was done as previously described (1). The multiple organ metastatic tissue micro array (TMA) was obtained from US biomax (MT2081).

Cell culture
Human triple-negative breast cancer cell line LM2-TGL-ZsGreen was derived from LM2-TGL cells (7,8) (a gift from the Massague lab, Memorial Sloan Kettering Cancer Center, New York, NY, USA) by retroviral infection with an MSCV-ZsGreen-2A-puro construct described previously (9). These cells were cultured in HyClone high-glucose Dulbecco’s modified Eagle medium (ThermoFisher Scientific), supplemented with 2 mM glutamine and 10% fetal bovine serum (Invitrogen) at 37°C in a 5% CO_2 incubator.

Two-photon imaging

NSG mice were injected with 2 x 10^6 LM2-TGL-Zsgr in their 4th right mammary fat pad. Since we were interested in testing the ability of the nanobodies to bind to primary tumors as well as to discrete micro metastases, we allowed the tumors to grow and metastasize for 6 weeks. Mice were injected with 10 µg of nanobodies labeled via sortase-mediated tagging with Alexa 647 (ThermoFisher Scientific). Two hours after nanobody injection, mice were euthanized, and their tumors and lungs were resected and imaged. Two-photon microscopy was done with an Olympus FV1000 MPE microscope fitted with a SpectraPhysics MaiTai DeepSee laser and 25 Å~ 1.05 NA water immersion objective with correction lens. Images were acquired using 840 nm excitation laser and filters 425/30 nm for second harmonic detection of collagen, 525/45 nm for Zs-Green, 607/70 nm for Texas red and 672/30 nm for Alexa 647. Images were acquired with the Fluoview FV10-ASW (version 4.1) software with a 5 µm Z resolution (512x512 pixel frames) to different axial depths within the tissues. Images were saved as Tiff files and scale bars were added using FIJI (version 1.0).

Synthesis of (Gly)3-Cys-NOTA

Synthesis was done using previously described methods (1,10). In brief, (Gly)3-Cys-NOTA was synthesized by dissolving maleimide-NOTA (Macrocyclics) in 0.1 M NaHCO3, pH 8.3 and adding the GGGC tetra-peptide at room temperature. Purification was done using reversed-phase HPLC on a preparative C18 column (Vydac, 218TP C18 column, 10 µm, 300 Å pore size, 2.2 x 250 mm) at a flow rate of 11.0 mL/min (solvent A, 0.05% TFA in H2O; solvent B, 0.043% TFA in 80% CH3CN). Fractions containing pure product were collected and lyophilized. MALDI-TOF MS calculated for C27H45N10O11S [M+H]+ was 717.298 (found = 717.213).

Enzymatic incorporation of (Gly)3-Cys-NOTA into nanobodies

Sortase tagging was used to site-specifically label NJT3, NJT4, NJT6, and NJB2 with GGGC-NOTA. The final products were confirmed by intact mass analysis. Approximately 5 pmol of each sample was loaded onto a C4 trap column (Michrom Bioresources) where the sample was desalted with aqueous HPLC buffer (0.1% formic acid). The desalted sample was subsequently introduced into the Qstar elite LC-MS/MS system (Applied Biosystems) using a fast gradient at a flow rate of 300 nL/min. BioTools software (QSTAR Analyst) was used to analyze the electrospray data.

Synthesis of ^{64}Cu-labelled nanobodies

Copper-64 ($^{64}CuCl_2$; radionuclide purity 98.5%) was produced in the MIR cyclotron facility at Washington University in St Louis. ^{64}Cu–nanobodies were synthesized and characterized as reported previously with minor modifications (1,10). In brief, in a typical reaction, NOTA conjugated nanobodies (30-90 uM solution in PBS) were incubated with 1.5-8 mCi of $^{64}CuCl_2$ at room temperature for 30-60 minutes in a 1.5ml eppendorf tube. The excess ^{64}Cu was removed by passing the mixture over a zeba spin desalting column (Thermofisher) followed by 2 washes with an Amicon 3,000-kDa MWCO filter (Millipore). The final products were analyzed by radio TLC. The ^{64}Cu-labeled nanobodies had a specific activity of 15.6-24.3 uCi/ug at the time of radiolabeling.

Radio TLC

Labeling of ^{64}Cu-labeled nanobodies was analyzed by radio TLC using iTLC-SA strips (Agilent), which consist of glass microfiber chromatography paper impregnated with a silicic acid (SA). Briefly, 1 ul of the mixture was applied to iTLC-SA strips and allowed to air dry for 5 minutes. The solvent (0.1 M Citrate buffer) was allowed to rise to 100 mm from the bottom of the strips. The strips were then dried and radiochemical purity was calculated by a TLC Scanner (Eckert and Ziegler AR2000 Imaging Scanner). The ^{64}Cu-labeled nanobodies were found to have a radiochemical purity of 83.5-92.18%.

Measurement of percentage of ^{64}Cu-Nanobodies retained in mice
NSG mice used for PET/CT imaging were anesthetized and injected i.v with ^{64}Cu-labelled nanobodies. After injection of radiolabelled nanobodies, the mice were placed in a cassette and inserted into a dose calibrator to measure the activity injected into the mouse. The mice were then allowed to wake up after injections and move around in their cages. Just before PET/CT imaging i.e ~ 2 hours after probe injection, the mice were scruffed to encourage them to urinate. Once again the activity retained in the mice was measured using the dose calibrator. Decay-corrected values were used to calculate the percentage of activity retained in the mouse 2 hours after injection.

Biodistribution analysis
Control and disease-bearing mice were euthanized ~150 minutes after injection of ^{64}Cu-labelled nanobodies. Blood was collected after cardiac puncture by inserting a syringe into the left ventricle. An incision was made to the right atria and mice were perfused with 20 mL PBS. After perfusion, multiple organs were resected and weighed in pre-weighed scintillation vials and placed in a gamma-counter (2480 Wizard2; PerkinElmer) to measure radioactive counts. To measure the uptake of ^{64}Cu-labelled nanobodies in different organs, decay and background-corrected counts were used to calculate the %ID/g for each tissue.

Statistical analysis
Graphpad prism (version 6.0) was used for statistical analysis and the following P values were considered: $P > 0.05$ (not significant), $P \leq 0.05$ (*), $P \leq 0.01$ (**), $P \leq 0.001$ (***) and $P \leq 0.0001$ (****). Statistical tests are indicated in the legends.

Supplementary table legends

Table S1
A. Complete MS data for TNBC patient samples including two independent biological duplicates from each of two patients with liver metastases, and two independent biological duplicates from each of two patients with lung metastases. All proteins identified are shown.
B. All detected matrisome proteins in TNBC patient samples from "A" are listed here.
C. Complete MS data for CRC patient samples including two independent biological duplicates from each of two patients with liver metastases. All proteins identified are shown.
D. All detected matrisome proteins in CRC patient samples from "C" are listed here.
E. Metastasis associated ECM signature of 67 matrisome proteins detected in all 6 patient samples that were represented by at least two peptides in one of the two biological duplicates from each patient.

The proteins listed in each tab are divided into Matrisome categories (core matrisome and matrisome associated) and further into indicated sub-categories. The following information is shared for each protein - entry name, entrez gene symbol, number of spectra observed, peptide abundance, and number of unique peptides identified.

Table S2
The 67 ECM proteins identified in all 6 patient samples as the "metastasis-associated signature" are ranked based on the mean precursor-ion intensities (of the two biological duplicates) corresponding to all peptides for a given protein for each sample. Collagens (blue text) and fibrinogen chains are highlighted (in grey). Several proteins are highlighted in the same color across different samples, including 5 proteins whose expression was confirmed by IHC in the CRC liver metastases samples in Fig 3D and Fig S3 (TGFBI, TNC, HSPG2, POSTN, EMILIN1) and protein FN1.

Table S3
IHC on mouse organs derived from NS mice with anti-TNC antibody (human and mouse specific) and NJT6-biotin (human specific). NJT6 did not bind to any normal mouse tissues, even though some tissues did stain with a rabbit monoclonal antibody to TNC. MM, muscularis mucosa; BM, basement membrane.

Supplementary References:

1. Jailkhani N, Ingram JR, Rashidian M, Rickelt S, Tian C, Mak H*, et al.* Noninvasive imaging of tumor progression, metastasis, and fibrosis using a nanobody targeting the extracellular matrix. Proc Natl Acad Sci U S A **2019**;116:14181-90
2. Ingram JR, Knockenhauer KE, Markus BM, Mandelbaum J, Ramek A, Shan Y*, et al.* Allosteric activation of apicomplexan calcium-dependent protein kinases. Proc Natl Acad Sci U S A **2015**;112:E4975-84
3. Naba A, Clauser KR, Ding H, Whittaker CA, Carr SA, Hynes RO. The extracellular matrix: Tools and insights for the "omics" era. Matrix Biol **2016**;49:10-24
4. Love MI, Huber W, Anders S. Moderated estimation of fold change and dispersion for RNA-seq data with DESeq2. Genome Biol **2014**;15:550
5. Guimaraes CP, Witte MD, Theile CS, Bozkurt G, Kundrat L, Blom AE*, et al.* Site-specific C-terminal and internal loop labeling of proteins using sortase-mediated reactions. Nat Protoc **2013**;8:1787-99
6. Rickelt S, Hynes RO. Antibodies and methods for immunohistochemistry of extracellular matrix proteins. Matrix Biol **2018**;71-72:10-27
7. Minn AJ, Gupta GP, Siegel PM, Bos PD, Shu W, Giri DD*, et al.* Genes that mediate breast cancer metastasis to lung. Nature **2005**;436:518-24
8. Ponomarev V, Doubrovin M, Serganova I, Vider J, Shavrin A, Beresten T*, et al.* A novel triple-modality reporter gene for whole-body fluorescent, bioluminescent, and nuclear noninvasive imaging. Eur J Nucl Med Mol Imaging **2004**;31:740-51
9. Stern P, Astrof S, Erkeland SJ, Schustak J, Sharp PA, Hynes RO. A system for Cre-regulated RNA interference in vivo. Proc Natl Acad Sci U S A **2008**;105:13895-900
10. Rashidian M, Keliher EJ, Bilate AM, Duarte JN, Wojtkiewicz GR, Jacobsen JT*, et al.* Noninvasive imaging of immune responses. Proc Natl Acad Sci U S A **2015**;112:6146-51
